# Molecular subtyping and prognostic modeling of colon adenocarcinoma based on programmed cell death features: a multi-omics and machine learning study

**DOI:** 10.3389/fimmu.2026.1736554

**Published:** 2026-06-11

**Authors:** Xiadiye Tuerhong, Yukai Zheng, Shihui Chen, Zheng Zhang, Yirixiati Aihaiti, Li Sun

**Affiliations:** 1 Department of Digestive Surgery, HongHui Hospital, Xi’an Jiaotong University, Xi’an, Shaanxi, China; 2 Department of General Surgery, The First Affiliated Hospital of Xi’an Jiaotong University, Xi’an, Shaanxi, China; 3 Department of Hepatobiliary Surgery, The First Affiliated Hospital of Xi’an Jiaotong University, Xi’an, Shaanxi, China

**Keywords:** colon adenocarcinoma, machine learning, molecular subtyping, multi-omics, predictive model, programmed cell death

## Abstract

**Background:**

Programmed cell death (PCD) plays a complex and critical role in the progression of colon adenocarcinoma (COAD). Elucidating PCD-related characteristics is expected to provide new insights for tumor subtyping, prognosis assessment, and personalized therapy.

**Methods:**

This study integrated multi-omics data from COAD and employed 10 clustering algorithms for molecular subtyping. Based on PCD-related genes, a Programmed cell death signature (PCDS) predictive model was constructed using 113 machine learning algorithms. The focus then shifted to the core gene of the model, TERT. Single-cell and spatial transcriptomic data were incorporated to decipher its cellular localization and regulatory pathways. Finally, the function of TERT was validated through *in vitro* experiments.

**Results:**

We categorized COAD into 5 molecular subtypes with distinct prognostic differences. Subsequently, we successfully developed an 18-gene PCDS. This model effectively predicted patient risk and overall survival in both the training set and multiple independent validation cohorts. The PCDS was closely associated with the tumor microenvironment, mutation burden, and response to immunotherapy. Single-cell and spatial transcriptomic analyses revealed that the core gene, TERT, was specifically highly expressed in malignant epithelial cells. *In vitro* experiments confirmed that knocking down TERT significantly inhibited the proliferation, migration, invasion, and clonogenic formation abilities of COAD cells. Mechanistically, TERT may inhibit apoptosis, regulate the cell cycle, and promote proliferation potentially through the E2F, G2/M checkpoint, and MYC signaling pathways.

**Conclusion:**

This study defines novel molecular subtypes of COAD through multi-omics clustering analysis and develops a robust PCD-related prognostic signature. Furthermore, it reveals the significant value of TERT as a potential therapeutic target in COAD.

## 1 Introduction

As the third most frequently diagnosed cancer worldwide, colorectal cancer (CRC) also ranks second in mortality among all malignancies (1). COAD is accountable for more than 90% of all diagnosed instances of colon cancer. It is the most common pathological subtype of colon cancer (2, 3). Despite the implementation of many management strategies such as surgery, chemotherapy, and endocrine therapy, the prognosis for patients with colon cancer remains unfavorable, with a five-year survival rate of only 67% (4, 5). Constructing a prediction model is required because of the intricate molecular landscape of CRC.

Most existing studies in colorectal cancer have relied on single-platform analyses, typically transcriptomic data alone (6). However, individual omics layers capture only partial aspects (7). For example, transcriptomic profiling cannot detect gene silencing through promoter hypermethylation, and mutational data alone do not reflect the transcriptional output that ultimately determines cell death pathway activity (8). Furthermore, non-coding RNAs such as miRNAs and lncRNAs are increasingly recognized as key regulators of PCD but are often overlooked in single-omics designs (9). Therefore, a multi-omics integration strategy is warranted to systematically characterize the subtypes in COAD across genomic, epigenomic, and transcriptomic dimensions, which may reveal subtypes and prognostic features that single-platform analyses would miss.

Programmed cell death (PCD) is a regulated process of intracellular self-destruction, crucial for maintaining tissue homeostasis and modulating disease progression. It is implicated in a wide spectrum of pathologies, including cancer, neurodegenerative disorders, and cardiovascular diseases (10, 11). Cancer cells can undergo various forms of cell death during tumor development. The ability to evade these cell death pathways is a recognized hallmark of cancer (12). Based on the initiating stimuli, cell death is broadly classified into two categories: accidental cell death, an uncontrolled and unregulated process, and PCD, which is tightly regulated and can be executed through diverse molecular mechanisms (13). The complicated interplay between PCD mechanisms and the pathogenesis of COAD has made them an important element of cancer biology. As of now, PCD can be divided into 15 subtype patterns, including pyroptosis, ferroptosis, necroptosis, autophagy, immunologic cell death, entotic cell death genes, cuproptosis, parthanatos, lysosome-dependent cell death, intrinsic apoptosis, extrinsic apoptosis, necrosis, anoikis, apoptosis-like morphology and necrosis-like morphology (14, 15). Apoptosis, which is the main kind of PCD, is closely linked to COAD and has an impact on the start, advancement, and reaction to treatment of tumors (16). In addition to apoptosis, various forms of PCD play an essential role in defining the CRC landscape. Pyroptosis, an extremely inflammatory form of cell death, has been discovered to control the microenvironment and immune responses in CRC (17). Ferroptosis, which occurs when lipids are peroxidized by iron independently, is linked to CRC development and resistance to conventional therapies (18). Different forms of PCD are linked through a complex web of initiation and signaling events that govern homeostasis, disease development, and treatment responses (19, 20). However, previous studies have only focused on individual PCD types. Therefore, it is essential to integrate all PCD modalities to identify a comprehensive set of signature genes, which is critical for developing robust markers for COAD treatment and prognosis.

We employed machine learning integration to develop an 18-gene PCDS that can accurately forecast the prognosis of COAD patients in the TCGA cohort, which was validated using five independent testing cohorts. Subsequently, we conducted a comprehensive investigation into the correlation between the PCDS and patient prognosis, immunological infiltration, and signaling pathways, thereby offering valuable insights into prognostication and the immune landscape in COAD. Furthermore, single-cell RNA sequencing analysis was employed to dissect the cellular heterogeneity of COAD and precisely map the expression of PCDS genes to specific cell populations, revealing their predominant localization in tumor cells. This resolution at the single-cell level provided a crucial context for interpreting the signature’s biological and clinical implications. Finally, we experimentally validated our findings.

## 2 Materials and methods

### 2.1 Multiomics data of COAD and data preprocessing of multicenter cohorts

We initially acquired multiomics data of COAD from the TCGA (https://portal.gdc.cancer.gov) COAD cohort. This data included complete transcriptome expression, DNA methylation, somatic mutations, and clinical information for the patients. The TCGAbiolinks package was utilized to obtain the transcriptome profiles of mRNA and lncRNA (21). The mature miRNA ID of TCGA was recorded using the miRBaseVersions.db software. The TCGAbiolinks tool was used to acquire the somatic mutations, which were then analyzed using the maftools program. The DNA methylation profile and clinical information were obtained from the UCSC Xena website (https://xenabrowser.net/). We also gathered comprehensive data on COAD from nine additional cohorts, which include six from the Gene Expression Omnibus (http://www.ncbi.nlm.nih.gov/geo)GEO: GSE39582, GSE33133, GS14333, GSE39084, GSE87211, GSE17536, and GSE29621. Additionally, we included one clinical trial from http://research-pub.gene.com/IMvigor210CoreBiologies, which is accessible under the Creative Commons 3.0 license (22). Transcripts per kilobase million (TPM) were obtained from the high-throughput sequencing of the transcriptome, and all array-based expression profiling was deduplicated and standardized.

Microarray expression profiles and corresponding clinical metadata were acquired from the official database via download. Raw expression data underwent background correction, log_2_ transformation, and quantile normalization using the limma package. The uniformity of sample expression abundance value distribution was assessed by visualizing the data using the boxplot function (23). These methods have been widely applied across multiple cancer types in prior research (24–28). For genes with multiple probes, the probe exhibiting maximal expression was selected as the representative gene expression value. Transcript expression was normalized to TPM values for RNA-seq analysis. This choice was based on the fact that TPM values exhibit more similarity to gene expression data obtained from microarrays. Additionally, using TPM values enhances the comparability of samples in our study (29). We applied limma’s removeBatchEffect function to integrate heterogeneous datasets. This function helps to correct for batch effects caused by non-biological technical biases in each dataset. The correction is done using an empirical Bayes framework (30, 31). Our approaches have been validated by numerous rigorous research, confirming the scientific integrity of the algorithms (32–34).To validate dataset integration efficacy, we performed Principal Component Analysis (PCA) on samples pre- and post-integration, following Liu et al.’s methodology (26). The single-cell RNA sequencing (scRNA-seq) dataset GSE166555 was retrieved from the GEO database, comprising paired tumor and adjacent normal samples from 12 patients (35). Quality control and cell filtering were performed using the “Seurat” package. The “Harmony” algorithm was applied to mitigate batch effects. Uniform Manifold Approximation and Projection (UMAP) was used for dimensionality reduction and visualization by clustering high-dimensional data. Cell annotation was performed with reference to the CellMarker database (http://xteam.xbio.top/CellMarker/index.jsp). Furthermore, this study utilized 10 CRC tissue sections from the Sparkle database (https://www.grswsci.top) for spatial transcriptome analysis, including 6 CRC samples (CRC, CRC2, CRC3, CRC4, CRC5, CRC6) and 4 CRC liver metastasis samples (CRC7, CRC8, CRC9, CRC10). Micro-regions were named according to the proportions of cell types present. We observed the average expression level of genes in micro-regions of different cell types across each section and applied the scale function for Z-score normalization to enhance comparability across different sections.

### 2.2 Multiomics consensus ensemble analysis

In order to conduct a thorough analysis, we initially aligned the omics data from the five dimensions using the sample ID (n = 386). The TPM expression data underwent a log2 transformation. We specifically chose probes that target CpG islands in the promoter region for our DNA methylation analysis. The mutation matrix comprised protein-altering variants including: frameshift indels, in-frame indels, nonsense, missense, nonstop, splice-site, and translation-start-site mutations.

We employed the “getElites” function from the MOVICS package to examine gene characteristics in this work. To identify the top 1,500 genes with the highest degree of variation among continuous variables such as mRNA, lncRNA, miRNA, and methylation, we utilized the “mad” method parameter in the “getElites” function. We performed Cox regression analysis integrating clinical covariates to identify statistically significant prognostic genes (p < 0.05) across all data dimensions. Using maftools’ oncoPrint, we first identified the top 5,000 most frequently mutated genes from binary mutation data. We further refined the gene selection by specifying method = “freq” to retain the top 5% most frequently mutated genes. The data obtained from these five dimensions were incorporated in our study for further investigation.

After initial feature selection, we determined the optimal cluster number for downstream analysis. It is widely recognized that the ideal number of clusters for data should be sufficiently small to minimize noise while still being large enough to preserve crucial information. We employed MOVICS’ getClustNum() function to determine optimal subgroup numbers, integrating the Clustering Prediction Index (CPI), Gap statistic, and Silhouette score (36, 37). Based on our previous studies on COAD, we classified it into five subtypes. We performed multi-omics clustering using MOVICS’ getMOIC function with 10 algorithms: CIMLR, ConsensusClustering, SNF, iClusterBayes, PINSPlus, moCluster, NEMO, IntNMF, COCA, and LRA. All methods were executed using default parameters via the methodslist argument. This gave us clustering results for each method. We applied MOVICS’ getConsensusMOIC function to integrate algorithm outputs via consensus clustering, enhancing robustness. Parameters were set as distance = “euclidean” and linkage = “average” (38). Final clusters were derived through this consensus integration.

### 2.3 Molecular profiling and stability of consensus subtypes

Pathway enrichment scores were computed using Gene Set Variation Analysis (GSVA) (30). Subsequently, the ESTIMATE R program was used to calculate the immune/stromal score of the tumor tissue. The GSVA method was used to assess the enrichment of 24 different types of immune cells inside the tumor microenvironment. To verify subtype stability, we first validated the clusters using subtype-specific biomarkers in the validation cohort. We then evaluated concordance between consensus clustering and both NTP and PAM classifiers.

### 2.4 Machine-learning algorithms developed a prognostic PPCDS

To identify the differentially expressed genes (DEGs) in COAD among the genes associated with PCD, we used the “limma” package with a threshold of |Log_2_FC| ≥ 1. After identifying potential prognostic biomarkers using univariate Cox analysis, we subjected these biomarkers to an integrative analysis procedure involving 10 machine-learning algorithms. These algorithms included random survival forest, elastic network (Enet), Lasso, Ridge, stepwise Cox, CoxBoost, partial least squares regression for Cox (plsRcox), supervised principal components (SuperPC), generalized boosted regression modeling, and survival support vector machine. Through this analysis, we were able to develop a prognostic clinical decision support tool that is both accurate and stable. The signature generation process comprised three stages (1): Prognostic biomarkers were identified by univariate Cox regression analysis of the TCGA dataset (2); Using these biomarkers, we developed prediction models through 113 algorithmic combinations, training them via leave-one-out cross-validation (LOOCV) within TCGA (3); All models were externally validated across five GEO cohorts (GSE14333, GSE29621, GSE17536, GSE39582, GSE33133).

### 2.5 Comparison with existing PCD−based prognostic models

To benchmark our PCDS against previously published PCD−related signatures for colorectal cancer, we selected three representative models: PCDscore (39), MPCDI (40), and PCDI (41). For each model, we retrieved the published gene lists and regression coefficients and calculated the risk score for each sample in the TCGA−COAD cohort using the same expression matrix. The concordance index (C−index) was computed using the survival R package to evaluate the discriminative ability of each model for overall survival.

### 2.6 Repeated cross−validation

A repeated 10−times 5−fold cross−validation was performed in the TCGA−COAD cohort to assess internal stability. In each repetition, the cohort was randomly divided into 5 folds, preserving survival event proportions. The PCDS model was trained on 4 folds and validated on the remaining fold. The C−index and time−dependent AUCs were calculated for each validation fold. The mean C−index across all 50 folds (10 repetitions × 5 folds) was reported with its standard deviation and 95% confidence interval.

### 2.7 Contribution analysis of PCD subtypes to the PCDS

To evaluate the relative contribution of different programmed cell death (PCD) subtypes to our 18−gene PCDS, we manually annotated each gene to the 15 PCD subtypes. For genes regulating cell cycle and proliferation, we assigned them to the apoptosis category because their dysregulation leads to mitotic catastrophe and apoptotic resistance. We then calculated the sum of absolute regression coefficients for genes belonging to each PCD subtype as a measure of its overall contribution to the risk score.

### 2.8 Model evaluation and nomogram construction

We visualized integrated gene expression profiles and clinical characteristics using a heatmap generated with the pheatmap R package. Stage distribution (I, II, III-IV) across risk strata was displayed via a ggplot2-generated stacked bar chart. To enhance survival prediction, we developed a nomogram (using the rms package) incorporating clinical variables (age, stage) and risk scores for 1-, 3-, and 5-year overall survival probability (31). Nomogram performance was validated through the receiver operating characteristic (ROC) curve (42), calibration curve, and concordance index curves.

### 2.9 Immune infiltration assessment

To comprehensively characterize tumor microenvironment (TME) heterogeneity across risk strata, we performed Hallmark gene set enrichment analysis (GSEA) using the clusterProfiler R package (33). Immune cell fractions were estimated using CIBERSORT (LM22 signature matrix, 100 permutations) and MCP−counter v1.0 on log_2_(TPM + 1) expression data and compared between risk groups by Wilcoxon test. Finally, we evaluated immunotherapy relevance by profiling immune checkpoint gene expression across risk groups using limma (34), correlating patterns with risk scores.

### 2.10 Mutational landscape analysis

We retrieved somatic mutation profiles for COAD patients from TCGA. Using the ComplexHeatmap R package, we visualized the mutational landscape after stratifying patients into four groups by median thresholds: H-TMB+high-risk, H-TMB+low-risk, L-TMB+high-risk, and L-TMB+low-risk, based on their median risk score and median tumor mutational burden. We retrieved somatic mutation profiles for COAD patients from TCGA. Using the ComplexHeatmap R package, we visualized the mutational landscape after stratifying patients into four groups by median thresholds. Survival differences across these strata were subsequently compared using Kaplan-Meier analysis.

### 2.11 Aneuploidy score calculation

Aneuploidy scores were derived from TCGA−COAD copy number segment data. A segment was considered altered if |log2 ratio| > 0.3. The aneuploidy score was defined as the fraction of the genome (hg19) affected by such alterations (43). Scores were compared between risk groups by Wilcoxon test.

### 2.12 Immunotherapy biomarker profiling

Immune checkpoints—critical regulators of immune activation—modulate immunological responses to prevent hyperactivation. We compared expression of established immune checkpoint genes (ICGs) between high- and low-risk groups and evaluated their correlations with model genes and risk scores. Additionally, COAD Immunophenoscores (IPS) were obtained from The Cancer Immunome Atlas (TCIA) (35) to assess immunotherapeutic vulnerability.

### 2.13 Enrichment analysis

We applied single-sample gene set enrichment analysis (ssGSEA) (36, 37)to quantify immune cell infiltration scores and evaluate immunological functions. We further employed the “clusterProfiler” and “org.Hs.eg.db” packages (38) to Analyze DEGs between risk groups and find enriched Gene Ontology (GO) concepts. The utilization of GSEA was also applied to ascertain the major enrichment of signaling pathways and biological activities in both the high- and low-risk groups (39).

### 2.14 Protein interaction network and core gene

We constructed a protein-protein interaction (PPI) network among prognostic signature genes using the STRING database (40). Moreover, patients were categorized into high- and low-expression groups based on the levels of risk genes expression in order to identify the core gene. Core genes were defined as those with degree centrality (number of direct interactions) above the 90th percentile in the PPI network, which was constructed using STRING with a confidence score ≥ 0.4. Although IGF1 exhibited the highest degree centrality, TERT was selected as the core gene because it directly interacted with IGF1 and WT1, its expression was significantly higher in TCGA-COAD tumors than in adjacent normal tissues, and it encodes the catalytic subunit of telomerase, which is essential for proliferation and apoptosis resistance. These criteria, together with its prognostic significance, justified TERT as the core gene for further validation.

### 2.15 Cell lines culture

The human COAD cell lines HCT116 and SW620 were acquired from the Cell Resource Center of the Shanghai Institute of Life Sciences. All cell lines were authenticated and cultured according to the supplier’s recommendations. HCT116 and SW620 cells were cultured in DMEM (Gibco, USA) supplemented with 10% fetal bovine serum (FBS) and 1% penicillin-streptomycin (Gibco, USA) at 37 °C in a humidified atmosphere containing 5% CO_2_.

### 2.16 Cell transfection

TERT knockdown was performed using small interfering RNAs (siRNAs) targeting TERT (sequencesprovided in **Supplementary Table 1**). For transfection, cells were seeded in 6-well plates at 50% confluence and transfected with either negative control (NC) or TERT-specific siRNAs using Lipofectamine 3000 (Invitrogen, USA).

### 2.17 Cell counting kit-8 experiment

Cells were seeded in 96-well plates at a density of 1×10³ cells per well. After adding 10 µL of CCK-8 reagent (A311-01, Vazyme) to each well, the plates were incubated at 37 °C in the dark for 2 hours. Cell viability was assessed by measuring the absorbance at 450 nm using a microplate reader (A33978, Thermo) at 0, 24, 48, 72, and 96 hours.

### 2.18 Colony formation

Cells were seeded into 6-well plates at a density of 1×10³ cells per well and cultured for 7 days. After rinsing twice with PBS, the cells were fixed with 4% paraformaldehyde for 15 minutes and subsequently subjected to Crystal Violet staining (Solarbio, China).

### 2.19 Wound-healing assay

Cells that had been genetically modified were placed in 6-well plates and grown until they covered 95% of the plate’s surface. An aseptic technique was used to create a solitary linear incision in each well using a 20-L plastic pipette tip, and detached cells and waste material were delicately rinsed out with PBS. The breadth of the scratch wounds was quantified using Image J software by capturing photographs at 0 and 48 hours.

### 2.20 Flow cytometry analysis of apoptosis

Apoptosis was assessed using the Annexin V−FITC/PI apoptosis detection kit (BD Biosciences, USA). After 48 hours of transfection with si−TERT or si−NC, HCT116 and SW620 cells were harvested, washed twice with cold PBS, and resuspended in 1× binding buffer at a concentration of 1 × 10^6^ cells/mL. Cells were then stained with 5 μL of Annexin V−FITC and 5 μL of propidium iodide (PI) for 15 minutes at room temperature in the dark. Apoptotic cell populations were analyzed within one hour using a flow cytometer (BD FACSCanto II, BD Biosciences). Early apoptotic cells (Annexin V^+^/PI^-^) and late apoptotic/necrotic cells (Annexin V^+^/PI^+^) were quantified. Each experiment was performed in triplicate, and data were analyzed using FlowJo software (TreeStar, USA). The percentage of total apoptotic cells (early + late) was compared between si−TERT and si−NC groups using an unpaired two−tailed t−test.

### 2.21 Western blot

Total protein was extracted from SW620 and HCT116 cells, and the protein content was determined by BCA kits (Beyotime, Shanghai, China). Samples with the same protein content were boiled for 10 min, transferred to SDS–PAGE, cut according to the molecular size markings of the target protein, sealed overnight, washed with primary antibody, incubated with primary antibody, washed with secondary antibody, and visualized by enhanced chemiluminescence (ECL) reagents. The results were analyzed by a Tanon chemiluminescence imaging analysis system. The TERT antibody was purchased from Shenzhen Youpin Biotechnology Co., Ltd. (Shenzhen, China). β-Tubulin antibody was purchased from Beyotime Biotechnology Co., Ltd. (Shanghai, China).

### 2.22 Statistical analysis

The R 4.2.0 program and the Sparkle online bioinformatics platform (https://www.grswsci.top) was used for data processing, statistical analysis, and visualization. The appropriate threshold was established using the “survminer” R package, and the survival program was used to perform Kaplan-Meier analysis (31, 35). The model’s accuracy was evaluated by using a ROC curve created via the use of the “timeROC” R package. Significant quantitative differences between regularly distributed variables were determined using a two-tailed t-test or a one-way ANOVA. For non-normally distributed data, a Wilcoxon test or a Kruskal Wallis test was used. Pearson’s correlation coefficients were used to analyze the correlations between two continuous variables. A significance threshold of P < 0.05 was used.

## 3 Results

### 3.1 Consensus molecular subtypes of COAD defined by multi-omics prognostic profiles

**Figure 1:**
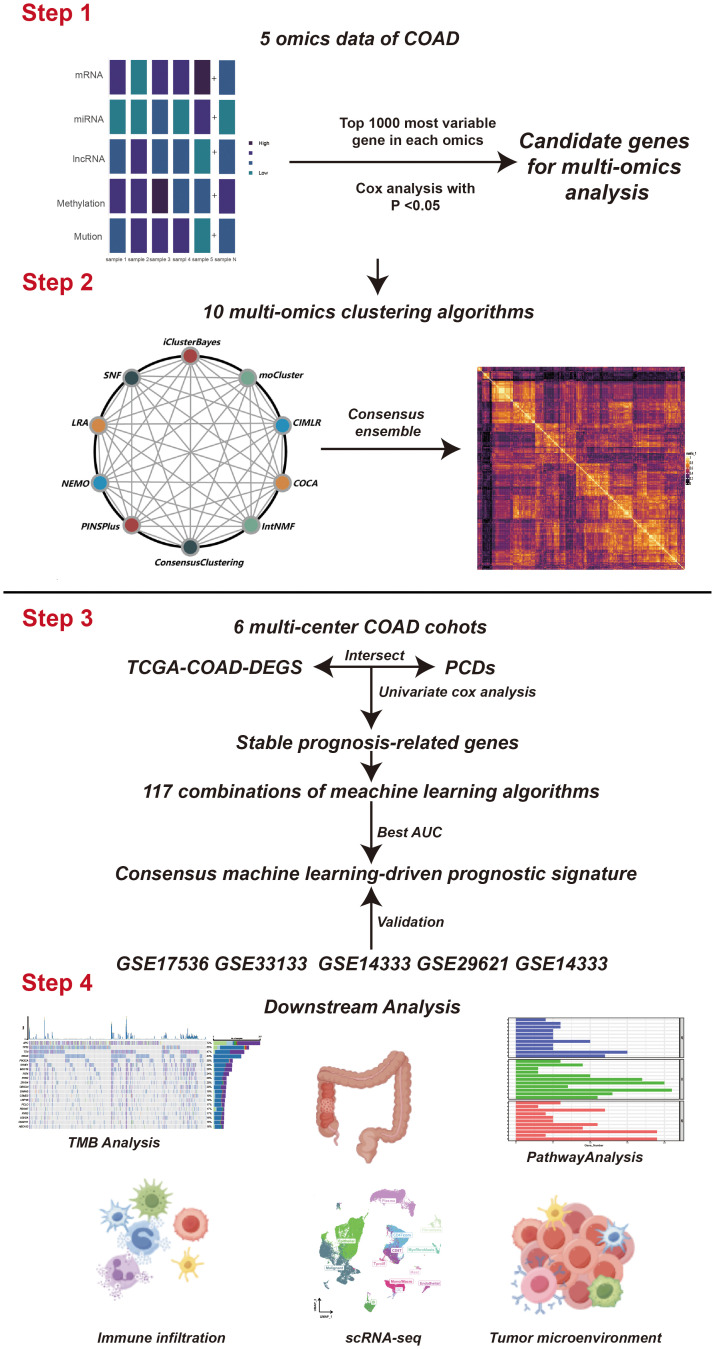
The research workflow for establishing the PCDS.

**Figure 2:**
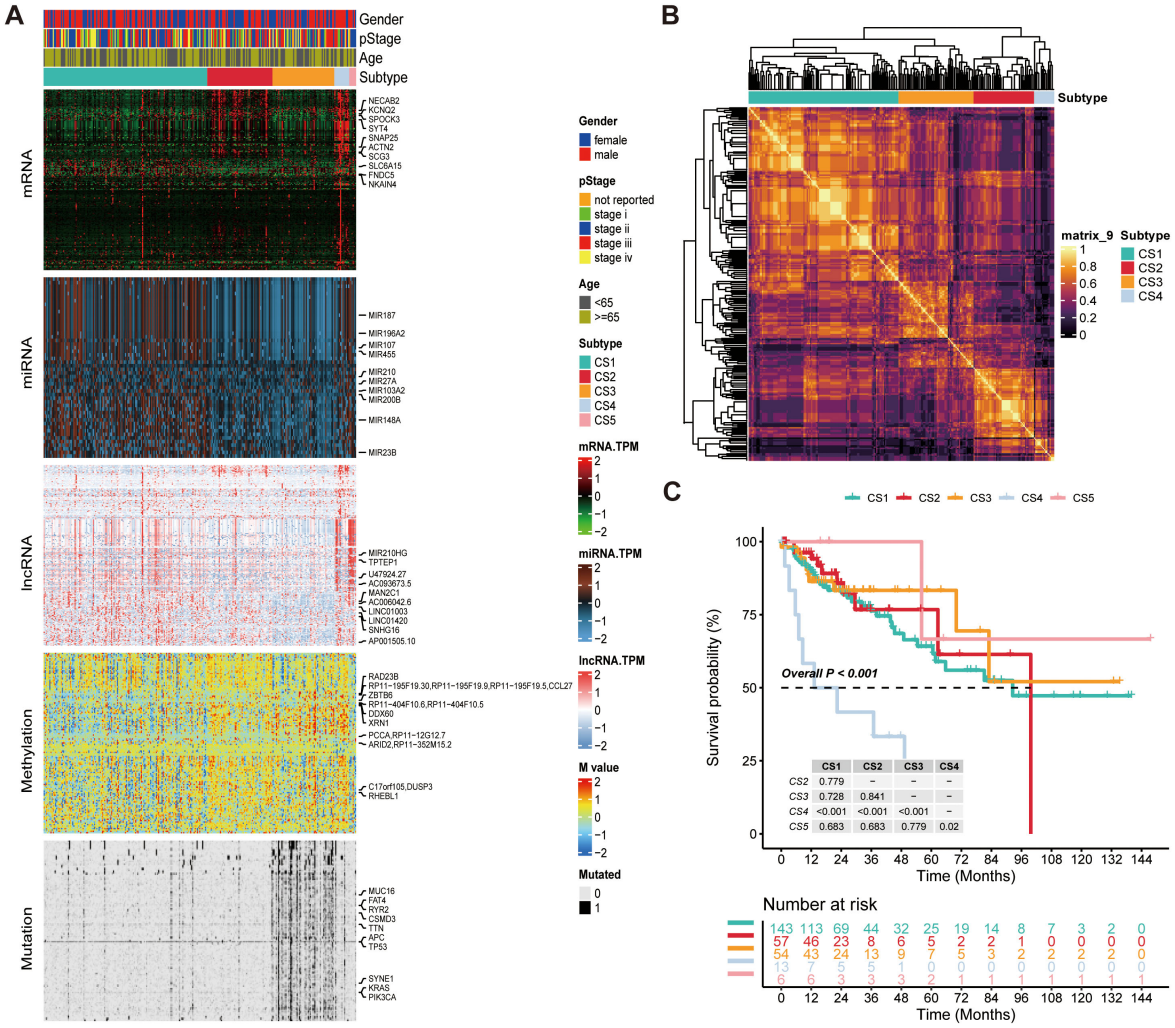
The multiomics integrative consensus subtypes of COAD. **(A)** Comprehensive heatmap of consensus ensemble subtypes, including mRNA, lncRNA, miRNA, DNA CpG methylation site, and mutant gene. **(B)** Consensus clustering matrix for five novel prognostic subtypes based on the 10 algorithms. **(C)** Different survival outcomes among the five subtypes.

The research workflow is shown in **Figure 1**. Upon conducting a thorough study of the data both before and after addressing the batch impact, we validated our findings by utilizing a PCA (**Supplementary Figures 1A, B**). We conducted a thorough examination of five subtypes using 10 multiomics ensemble clustering techniques. Our decision-making process was based on the cluster prediction index, gap statistical analysis, silhouette score, and prior research experience (**Supplementary Figures 2A, B**). Subsequently, clustering outcomes were integrated through a consensus ensemble approach, incorporating distinct molecular expression profiles from multi-transcriptomic layers (mRNA, lncRNA, and miRNA), epigenetic methylation patterns, and somatic mutation landscapes. (**Figures 2A, B**). The correlation between our categorization method and overall survival (OS) was significant (p < 0.001; **Figure 2C**). Cancer subtype 3 (CS3) had the most advantageous survival results compared to all other assessed subtypes.

Using the omics data from each dimension, we assessed the top 1,000 genes with the highest degree of variation. We then identified candidate prognostic genes for clustering through Cox regression analysis, prioritizing those with significant associations with patient survival outcomes. In analyzing mutation data, we assessed potential genes by considering the prevalence of mutations. Consensus clustering was used to obtain stable COAD prognostic subtypes. This was achieved by applying 10 different multiomics clustering algorithms, including iClusterBayes, moCluster, CIMLR, IntNMF, ConsensusClustering, COCA, NEMO, PINSPlus, SNF, and LRA.

### 3.2 Partitioning of COAD integrative consensus molecular subtypes

**Figure 3:**
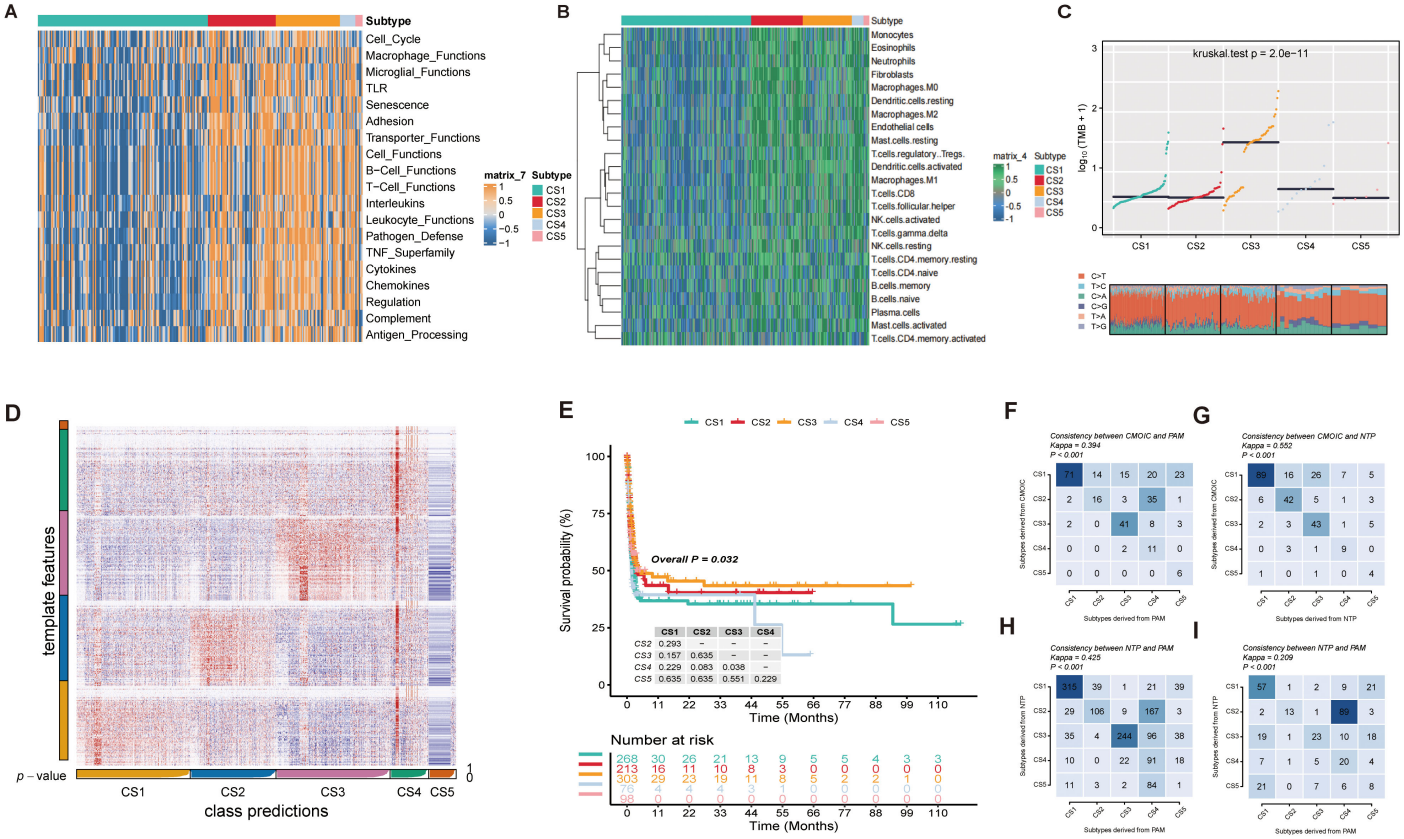
Molecular landscape and validation of COAD CSs. **(A)** The enrichment of five subtypes by ssGSEA. **(B)** Immune profiles in the TCGA-COAD cohort. **(C)** The TMB distribution among the five subtypes. **(D)** Validation of COAD CSs in the nearest template of the META-COAD cohort. **(E)** Survival analysis of COAD CSs in the META-COAD cohort. **(F)** The consistency of CSs with NTP in the TCGA-COAD cohort. **(G)** The consistency of CSs with PAM in the TCGA-COAD cohort. **(H)** The consistency of NTP with PAM in the META-COAD cohort. **(I)** The consistency of NTP with PAM in the IMvigor-COAD cohort.

Currently, molecular classification of COAD is based on distinct molecular expression profiles, which are often linked to specific biological processes and may inform prognostic and therapeutic strategies. Thus, we conducted a thorough analysis of the various molecular characteristics of these CSs. Through the application of the ssGSEA algorithm, it was discovered that the sample contained a wide range of molecular signatures that were undergoing enrichment. It is worth noting that we discovered significant enrichment of pathways related to cell cycle and senescence in CS2 and CS3. On the other hand, pathways associated with cell function were significantly enriched in CS1. This suggests that CS2 and CS3 may play a role in cell proliferation and division, while CS1 is more focused on cellular regulation (**Figure 3A**).

Recognizing the critical role of tumor immunity in tumorigenesis and progression, we assessed immune cell infiltration within the tumor microenvironment and observed significantly elevated levels in subtypes CS2, CS3, CS4, and CS5. On the other hand, CS1 exhibited a comparatively lower level of infiltration (**Figure 3B**).

We then analyzed the Mutational landscape of the five subtypes and showed that CS3 had the highest TMB, suggesting that the more neoantigens are likely to be produced by CS3, the higher the tumor immunogenicity, Highlighting the greater advantage of undergoing treatment with PD-1/PD-L1 immune checkpoint inhibitors (ICIs) (**Figure 3C**).

To investigate the reaction of these five subtypes to medications, we selected five commonly used chemotherapeutic agents for CRC and found that these five subtypes responded differently to different chemotherapeutic agents (**Supplementary Figure 3**). This result informs the choice of subsequent chemotherapeutic agents for CRC.

The external cohort samples are categorized into one of the identified CSs using the closest template prediction (NTP) method. According to these results, the CS3 subtype in the META-COAD cohort, which was determined using data from 6 cohorts, had the highest survival rate compared to other subtypes. The statistical significance (p < 0.005) was consistent across various external cohorts (**Figures 3D, E**). The CSs’ consistency with NTP and the partition around medoids (PAM) algorithms was assessed as well (p < 0.005; **Figures 3F–I**).

### 3.3 Development of the PCDS

**Figure 4:**
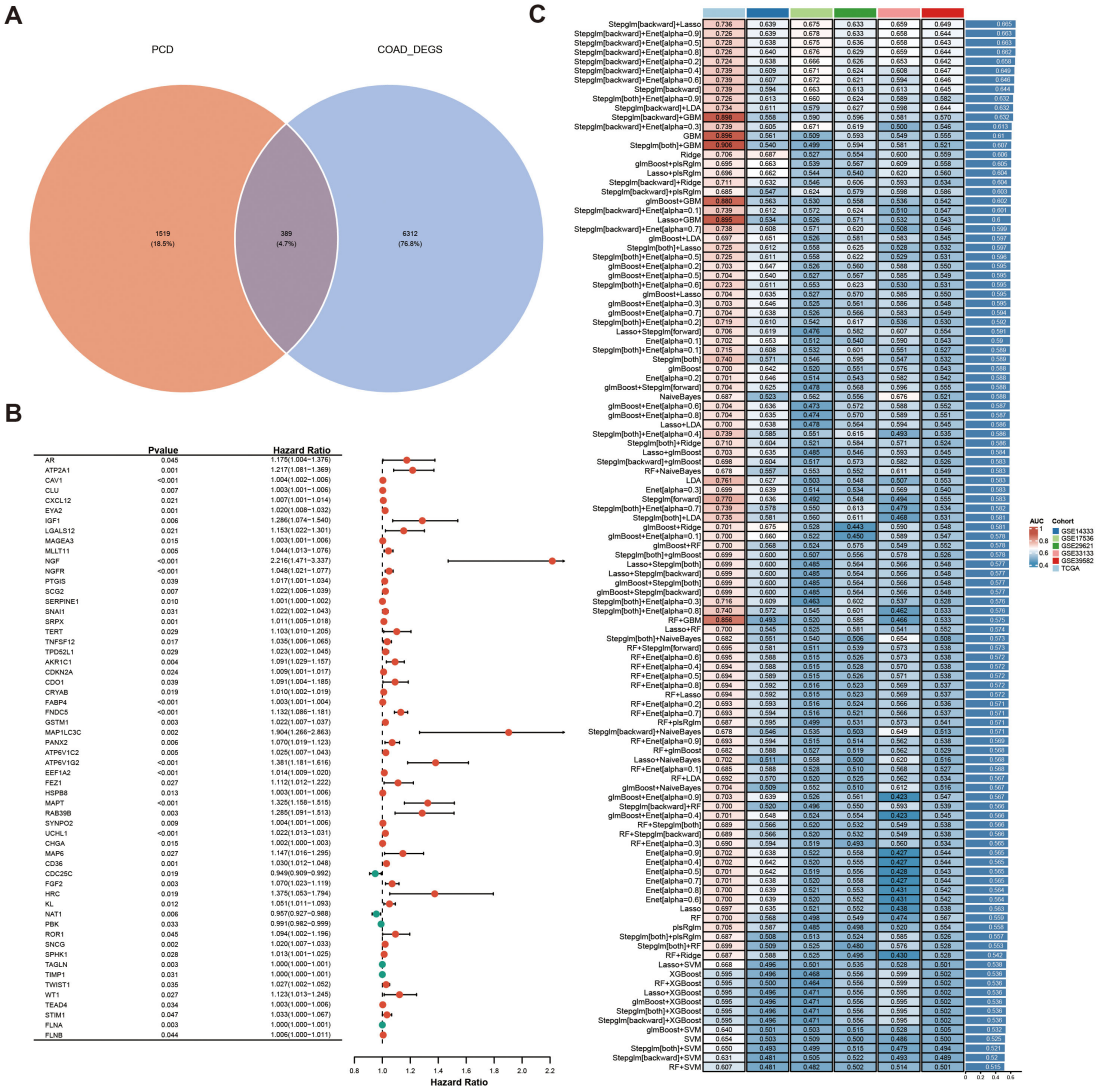
Construction of the PCDS. **(A)** Venn plots identified the genes most associated with PPCDS. **(B)** Screening for prognostically relevant PPCDS by univaricox analysis **(C)** A total of 117 kinds of prediction models via LOOCV framework and further calculated the C-index of each model across all validation datasets.

Among 1908 PCD -related genes, we identified 389 DEGs in COAD using thresholds of |log_2_FC| ≥ 1 and an adjusted p-value < 0.05. (**Figure 4A**). A total of 61 genes were discovered to have a significant connection with the prognosis of COAD among the identified genes (**Figure 4B**).

In order to make sure our prognostic PCDS is accurate and stable, we used an integrated machine-learning procedure that includes the 10 methods mentioned above to submit these 61 potential prognostic biomarkers. At the end, we discovered a total of 113 different types of prognostic models and their C-index in the training and testing cohort (**Figure 4C**). The stepglm [forward] + Lasso method model, which had the highest average C-index of 0.665, was deemed to be the optimal prognostic model (**Figure 4C**; **Supplementary Figure 4**). Comparison with existing PCD related prognostic models. We benchmarked our PCDS against three published PCD based signatures (PCDscore, MPCDI, PCDI) in the TCGA COAD cohort. Our PCDS achieved the highest C index (0.736, from leave one out cross validation), outperforming PCDscore (0.632), PCDI (0.560), and MPCDI (0.530) (**Supplementary Figure 5**). To further evaluate the internal stability and potential overfitting of our PCDS model, we performed a repeated 10 times 5-fold cross validation within the TCGA COAD cohort. The model achieved a mean C index of 0.71 (SD = 0.05) across all 50 validation folds, with a 95% confidence interval of 0.68–0.74 (**Supplementary Figure 6**). These results indicate that the PCDS model exhibits reasonable internal stability and does not suffer from severe overfitting, supporting its robustness within the TCGA dataset. The prognostic PCDS score was derived using 18 genes associated with PCD, and the PCDS score of every COAD patient was determined by the formula: risk score = (0.071362465) ×ATP2A1 +(0.252562595)×IGF1 + (-0.017343164)×PTGIS + (0.001624859)×SERPINE1 + (0.020188644 )×SRPX + (0.131860601)×TERT + ( 0.159571899)×FNDC5+ (0.023136528)×GSTM1 + ( 0.026909359)×ATP6V1C2 + (0.021145164)×EEF1A2 + (-0.453082006) ×RAB39B + (0.010507800) ×SYNPO2 + (-0.541040856) ×MAP6 + (-0.061617697) ×CDC25C + (0.021081521) ×KL +(0.000455843)× TIMP1 + (0.008889071) × WT1 + (0.004602129) × TEAD4 (**Supplementary Table 2**). The prognosis of these 18 genes was analyzed in the TCGA database (**Supplementary Figure 7**). To determine whether all programmed cell death (PCD) subtypes contribute equally to the 18 gene CDS or whether a subset drives the signature, we quantified the sum of absolute coefficients for each PCD subtype. Apoptosis−related genes contributed 62.0% of the total absolute coefficient sum, autophagy−related genes contributed 38.0%, and all other 13 PCD subtypes (e.g., pyroptosis, ferroptosis, necroptosis, cuproptosis) contributed less than 1% and were negligible (**Supplementary Figure 8A**). Positive coefficients (risk factors) were predominantly observed among apoptosis−related genes such as IGF1, TERT, and FNDC5, whereas negative coefficients (protective factors) were mainly associated with autophagy−related genes including MAP6 and RAB39B (**Supplementary Figure 8B**). These results indicate that the CDS is primarily driven by apoptosis and autophagy rather than by all PCD modalities equally.

### 3.4 Survival analysis and model evaluation

**Figure 5:**
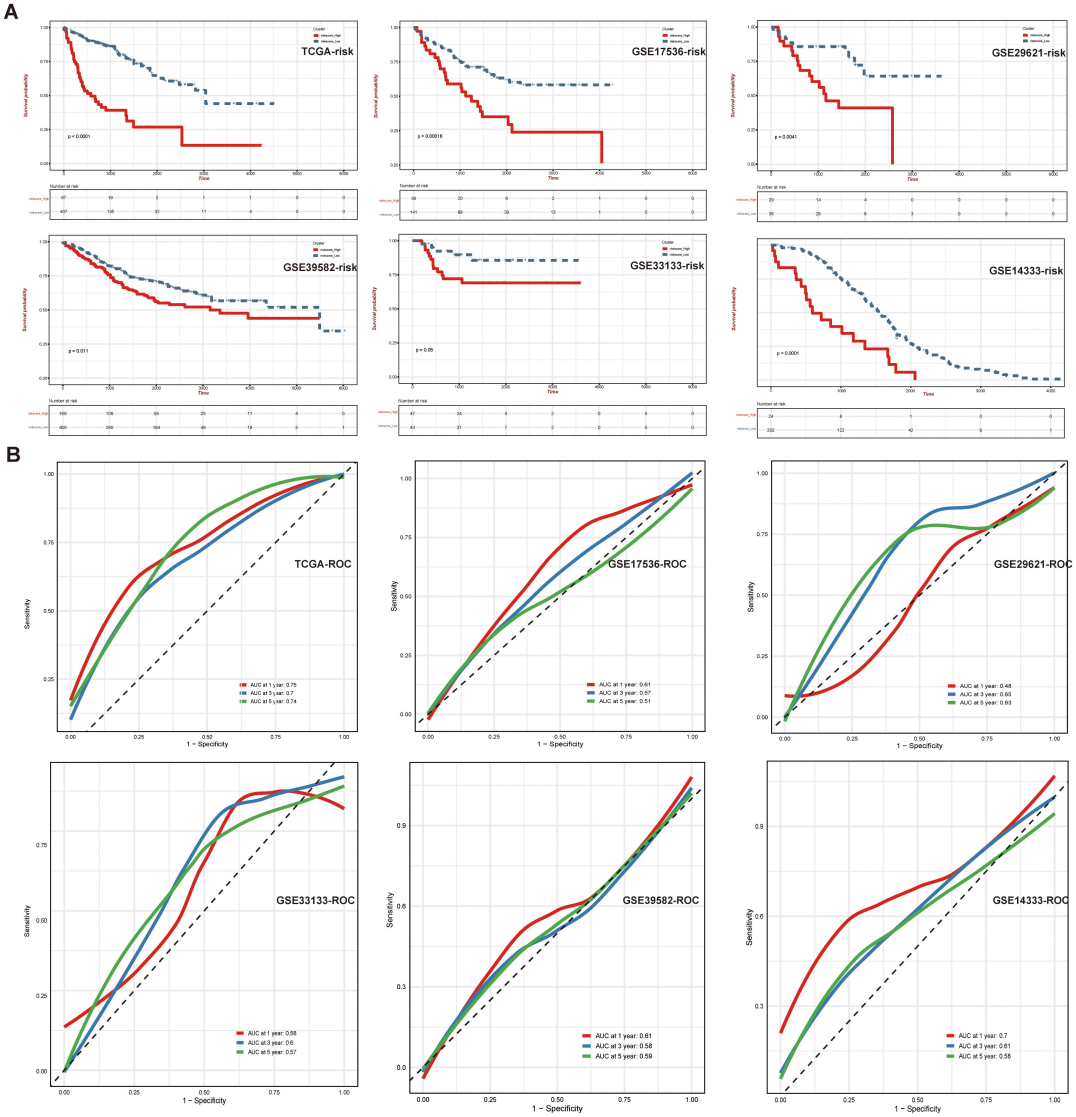
Assessment of risk models. **(A)** Kaplan-Meier survival analysis of signatures in the TCGA and five GEO datasets. **(B)** The ROC curve was used to evaluate the performance of the model in the TCGA and five GEO datasets.

Patients were classified into high- or low-risk groups based on a predetermined cutoff value of the risk score. Significant differences in OS were observed between these groups across both the TCGA-COAD cohort and five independent GEO datasets. (**Figure 5A**). The discriminative ability of the PCDS was evaluated through ROC analysis, yielding AUCs of 0.75, 0.7, and 0.74, for TCGA-COAD at 1-, 3-, 5-year time points, respectively. The AUCs for GSE17536 were 0.61, 0.57, and 0.51, while for GSE29621, the AUCs were 0.48, 0.65, 0.702, and 0.63. In the case of GSE33133, the AUCs were 0.58 0.6, and 0.57 and for GSE39582, the AUCs were 0.61 0.58, and 0.59. Finally, for GSE14333, the AUCs were 0.7, 0.61, and 0.58, respectively (**Figure 5B**). In addition, some commonly used indicators related to prognosis such as gender, age, staging, etc. and risk scores were included in the multifactorial cox analysis in the six datasets. The results suggest that risk scores are associated with COAD prognosis (**Supplementary Figure 9**).

### 3.5 Construction and validation of prognostic nomogram

**Figure 6:**
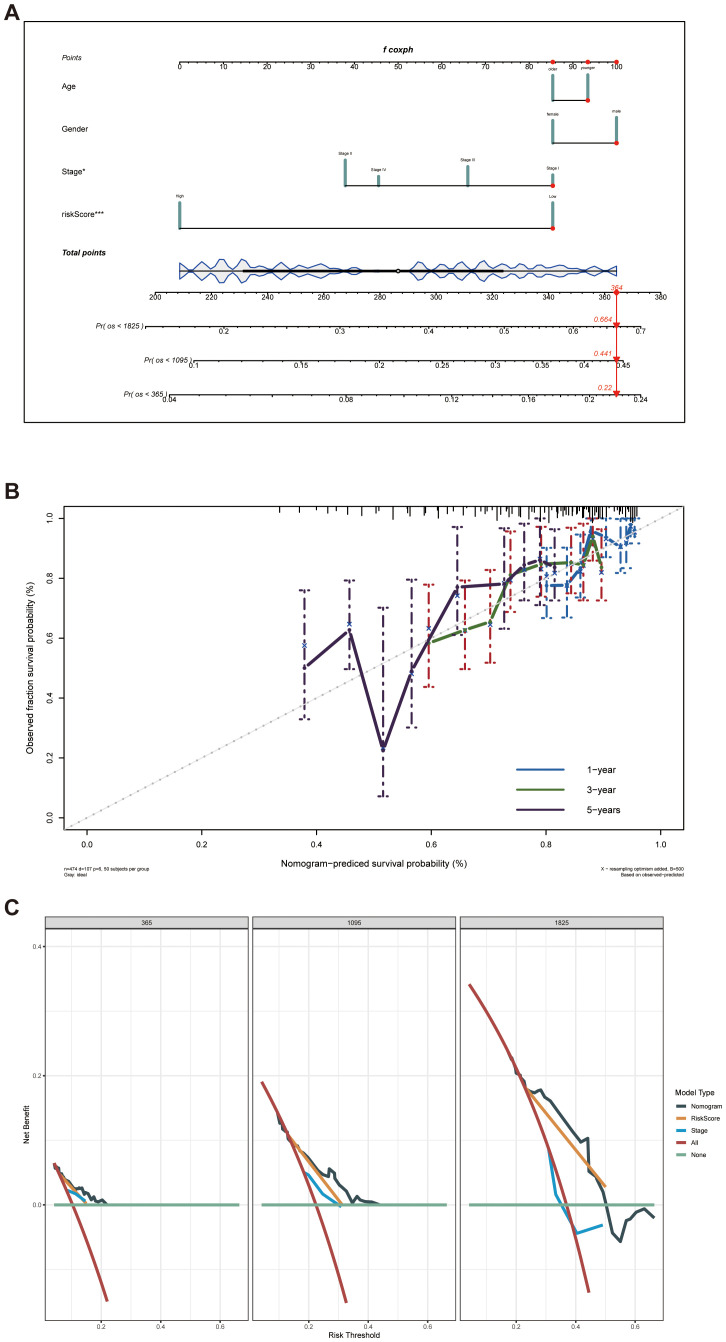
Developing an accurate nomogram. **(A)** A nomogram model was constructed to predict the 1-year, 3-year, and 5-year overall survival of ESCA patients. **(B)** Calibration curves of the nomogram model for 1-year, 3-year, and 5-year overall survival. **(C)** Decision curve analysis for 1-year, 3-year, and 5-year overall survival of the nomogram model.

Using the TCGA-COAD dataset, we created a predictive nomogram that considers the risk score and clinicopathological factors (gender, age, and clinical stage) in order to enhance prognosis prediction (**Figure 6A**). Clinical outcomes, including 1-, 3-, and 5-year survival status, were evaluated as prognostic endpoints. The calibration plot indicated that the PCDS exhibited excellent predictive accuracy for survival probabilities at each time point. (**Figure 6B**). Based on the C-index analysis, the nomogram exhibited significantly better prognostic performance compared to the risk score alone and all other individual clinical parameters. (**Figure 6C**).

### 3.7 Differences in the immune microenvironment and immunotherapy response

**Figure 7:**
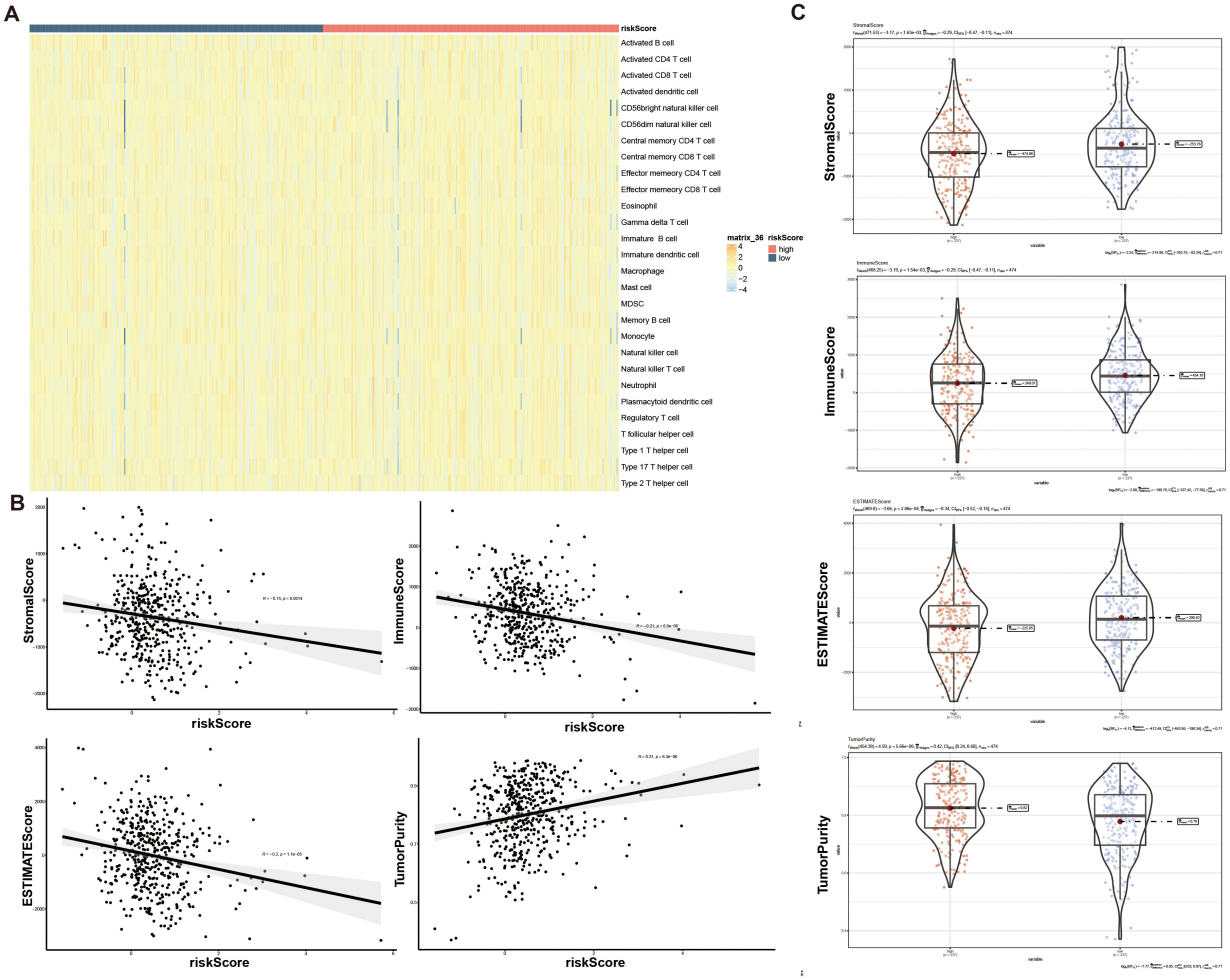
Analysis of immune infiltration. **(A)** ssGSEA algorithms assess differences in immune infiltration status between different risk groups. **(B)** The correlations in stromal score, immune score, ESTIMATE score, and tumor purity calculated using the ESTIMATE algorithm between the two risk subgroups. **(C)** The violin plot demonstrated the difference in stromal score, immune score, ESTIMATE score, and tumor purity calculated using the ESTIMATE algorithm between the two risk subgroups.

**Figure 8:**
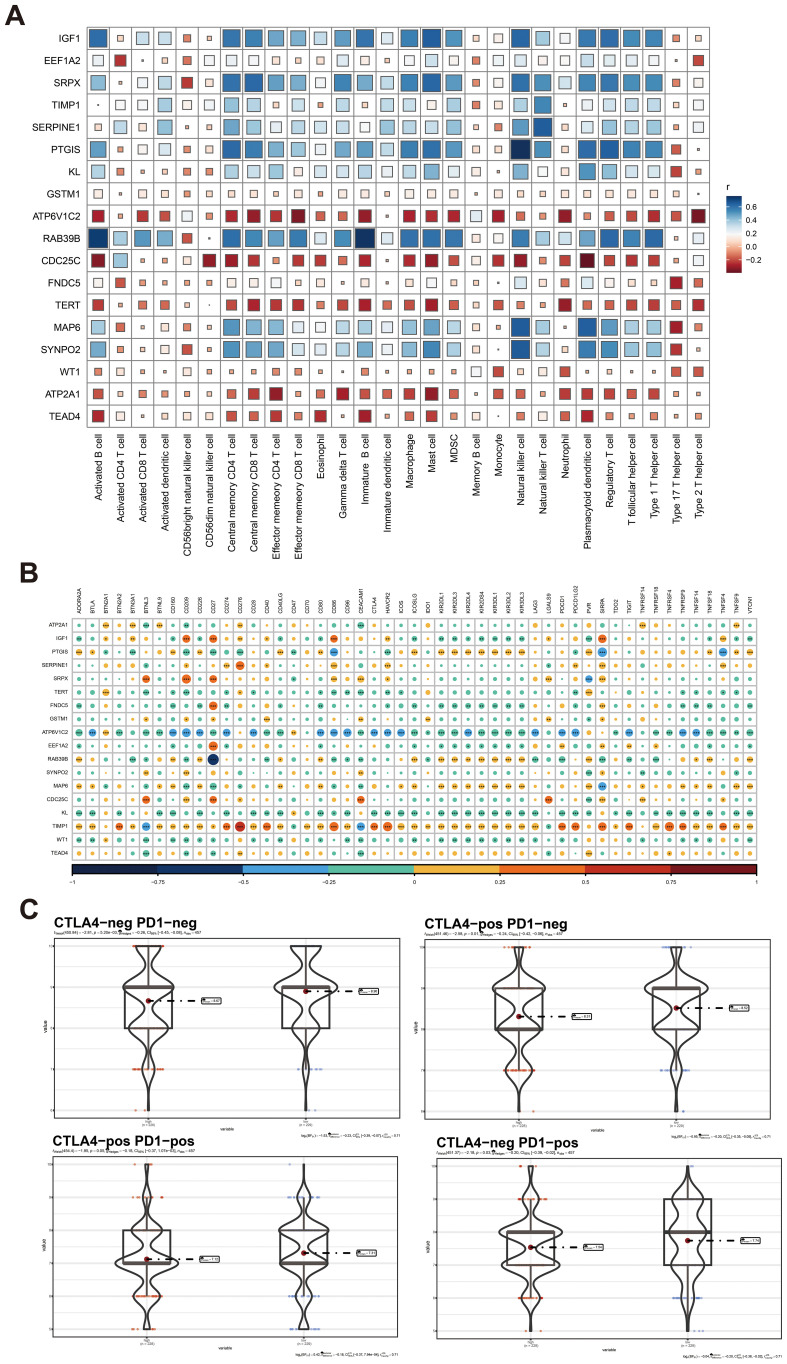
Immune checkpoint and TCIA analysis. **(A)** A heatmap showed the correlation between risk gene expression and 22 types of immune cells. **(B)** Correlation between model genes and immune checkpoint. **(C)** The low-risk group has significantly greater IPS, IPS-CTLA4-neg-PD-1-neg, IPS-CTLA4-pos-PD-1-neg, IPS-CTLA4-neg-PD-1-pos, and IPS-CTLA4-pos-PD-1-pos. *P < 0.05, **P < 0.01, ***P < 0.001.

Through the application of ssGSEA algorithms, it was observed that high-risk tumors had greater infiltration of T cells, B cells, NK cells, and activated mast cells (**Figure 7A**). Risk score was negatively correlated with stromal (R = -0.15), immune (R = -0.21), and ESTIMATE scores (R = -0.20), and positively correlated with tumor purity (R = 0.21, all FDR < 0.001); the low-risk group showed higher stromal, immune, and ESTIMATE scores (**Figures 7B, C**). CIBERSORT and MCP−counter analyses further confirmed that the low−risk group had significantly higher fractions of plasma cells, resting memory CD4^+^ T cells, B cells, myeloid dendritic cells, and CD8^+^ T cells, while the high−risk group showed higher M0 macrophages (**Supplementary Figure 10**). We further evaluated the association of the risk score with immunotherapy biomarkers. The relationships between risk genes and 22 immune cell types, as well as between model genes and immune checkpoint genes (ICGs) (**Figures 8A, B**). IPS analysis indicated that low-risk patients had significantly higher IPS scores, suggesting a greater likelihood of benefiting from anti−PD−1/CTLA−4 therapy (**Figure 8C**). To characterize the immune landscape of the low- and high-risk groups beyond immune cell infiltration, we systematically compared effector/checkpoint ratios and exhaustion marker expression between the two risk strata. None of the effector/checkpoint ratios (GZMB/PDCD1, IFNG/CTLA4, PRF1/LAG3, GZMA/TIGIT) differed significantly between the low- and high-risk groups. However, TIGIT and TOX expression levels were significantly higher in the low-risk group, whereas other canonical exhaustion markers such as PDCD1, CTLA4, LAG3, and HAVCR2 showed no significant differences (**Supplementary Table 3**). These findings suggest that the low-risk group does not exhibit a classical exhausted phenotype; rather, the elevated TIGIT and TOX expression may reflect an activated T cell state, as both molecules can be upregulated during early T cell activation. Furthermore, we calculated aneuploidy scores for each sample. The high−risk group had significantly higher aneuploidy scores than the low−risk group, indicating greater genomic instability that may contribute to immune evasion (**Supplementary Figure 11**).

### 3.8 Mutational landscape

**Figure 9:**
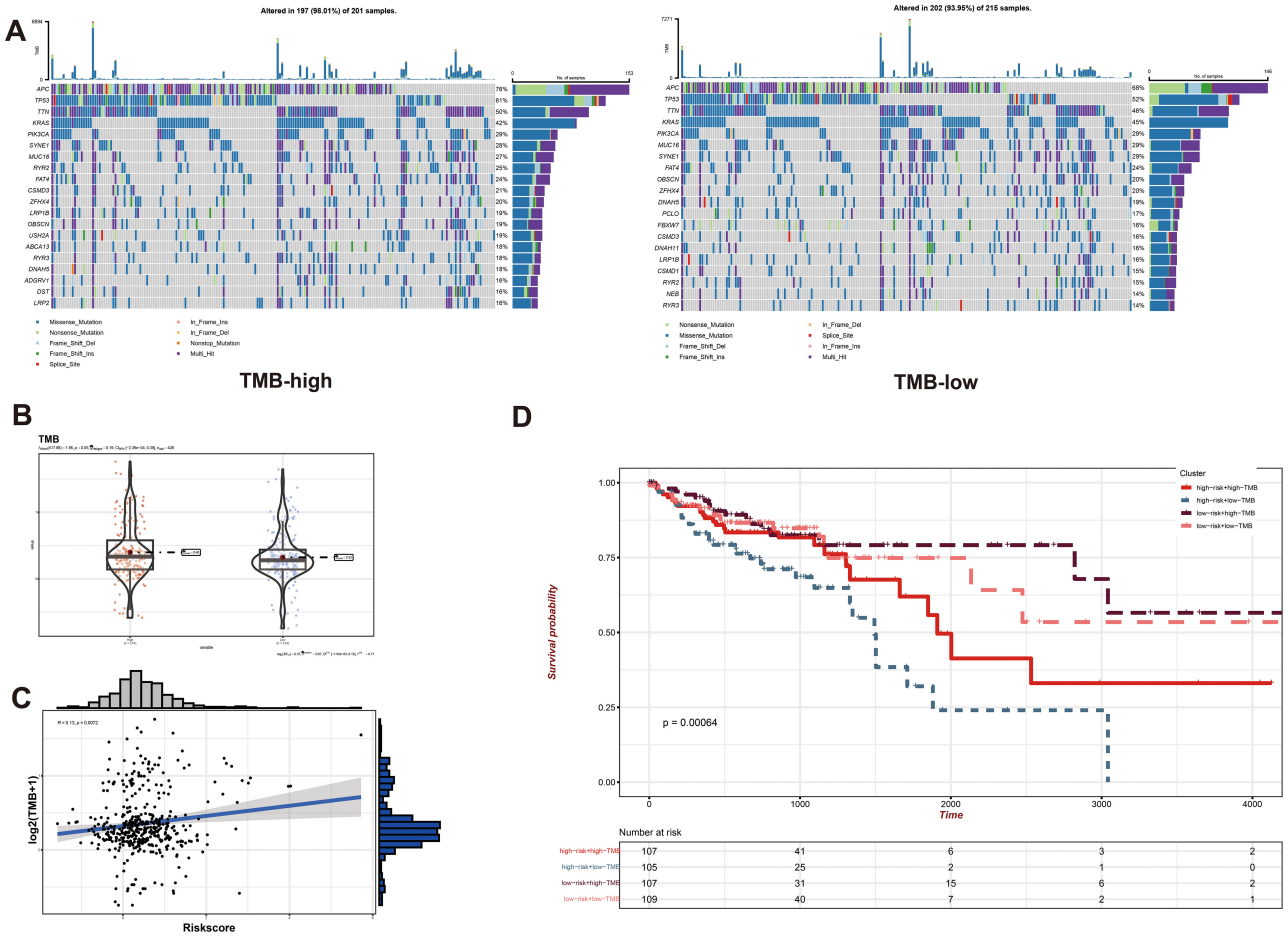
Landscape of LUAD sample mutation profiles. **(A)** Mutation landscape of the top 20 genes with mutation frequency in differential risk subgroups. **(B)** Comparison of tumor mutation burden (TMB) between different risk groups. **(C)** Correlation analysis between risk score and TMB. **(D)** Survival differences for four different subgroups (H-TMB+high-risk, H-TMB+low-risk, L-TMB+high-risk, and L-TMB+low-risk).

The high-risk group had a higher incidence of gene mutations, including APC, TP53, and TTN (**Figure 9A**), and exhibited a significantly higher tumor mutation burden (TMB) than the low-risk group (**Figure 9B**). Risk score was positively correlated with TMB (Spearman R = 0.13, P < 0.05, **Figure 9C**). When patients were stratified by median TMB and median risk score, the H−TMB/low−risk subgroup had the best prognosis, whereas the L−TMB/high−risk subgroup had the poorest survival (**Figure 9D**). Beyond the overall TMB difference, we further compared the mutation frequencies of individual genes between the two risk groups. Several genes showed nominally higher mutation rates in the high−risk group, including MET, AK5, NOTCH1, and TP73, while genes such as TTC21B and MED12 were more frequently mutated in the low−risk group (**Supplementary Figure 12**). Although these differences did not remain significant after multiple testing correction, they are consistent with the higher TMB observed in high−risk tumors.

### 3.9 Functional enrichment analysis

**Figure 10:**
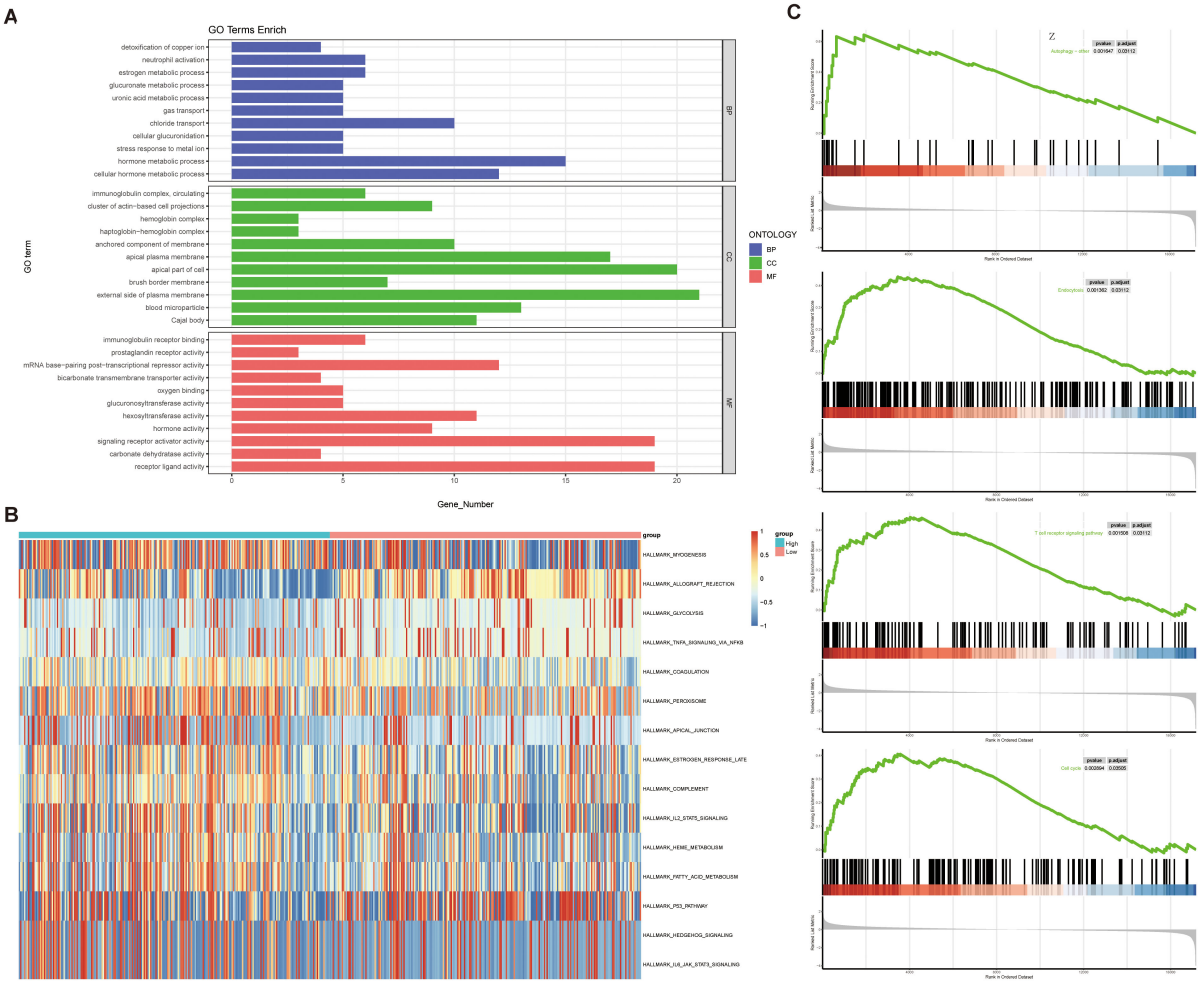
Enrichment analysis. **(A)** A bar plot showed GO enrichment analysis. **(B)** GO enrichment of GSVA between high- and low-risk groups. **(C)** GSEA showed pathway differences between high- and low-risk groups.

Pursuant to differential expression analysis, a discrepancy in the expression levels of genes was identified between the low- and high-risk groups (P < 0.05 and log_2_FC > 1) (**Supplementary Figure 13**). Subsequently, GO enrichment analysis was performed on the DEGs (**Figure 10A**). The results indicated that the top three significantly enriched Biological Process (BP) terms were cellular hormone metabolic process, hormone metabolic process, and neutrophil activation. Cellular Component (CC) included external side of plasma membrane, immunoglobulin complex, circulating, and apical part of the cell; and Molecular Function (MF) involved signaling receptor activator activity, hormone activity, and immunoglobulin receptor binding. GO enrichment of GSVA showed that Classical Tumor Pathways such as HALLMARK_IL2_STAT5_SIGNALING, HALLMARK_P53_PATHWAY was enriched in high-risk group (**Figure 10B**). Additionally, GSEA enrichment analysis showed that Autophagy, Endocytosis, T cell receptor signaling pathway and Cell cycle were primarily enriched in the high-risk group (**Figure 10C**).

### 3.10 Identification of TERT as a core oncogene and elucidation of its regulatory pathways

**Figure 11:**
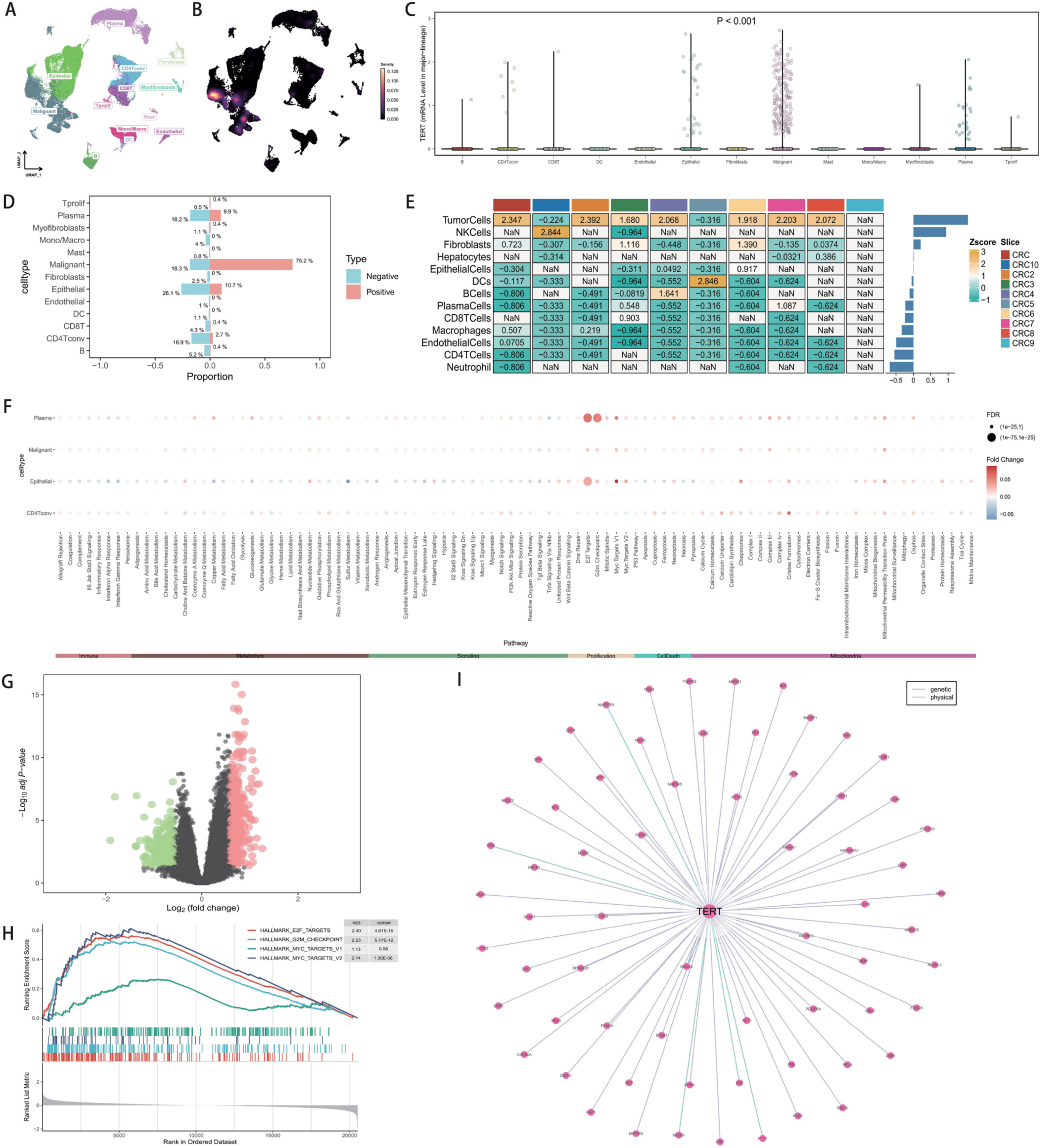
Cellular localization of TERT and its mediated regulatory pathways. **(A)** UMAP plot showing 13 distinct cell clusters after cell clustering and annotation of the GSE166555 dataset. **(B)** Cellular distribution of TERT. The UMAP plot reveals its predominant expression in malignant tumor cells. **(C)** Expression levels of TERT across different cell types. **(D)** Proportion of TERT-positive and TERT-negative cells within different cell types. **(E)** TERT expression scores in different cellular micro-regions across 10 spatial transcriptome sections. **(F)** The AUCell method was used to score various biological pathways in TERT-positive and TERT-negative cells, comparing the score differences between these groups within each cell type. **(G)** Volcano plot displaying the differential expression analysis for the TCGA-COAD dataset, comparing the top 30% of samples with the highest TERT expression against the bottom 30%. **(H)** Gene set enrichment analysis (GSEA) was performed on the differential expression results. Gene sets with a normalized enrichment score (NES) absolute value greater than 1 and an FDR-adjusted q-value less than 0.25 were considered significantly enriched. Comparison with the pathway scoring results from the GSE166555 single-cell dataset revealed that the E2F targets, G2/M checkpoint, and MYC signaling pathways were also significantly enriched in the TCGA-COAD cohort. **(I)** A TERT-centered protein-protein interaction network constructed using the BioGRID database, showing multiple proteins involved in the E2F, G2/M checkpoint, and MYC signaling pathways interacting with TERT.

We utilized the STRING database to generate a protein-protein interaction (PPI) network (**Supplementary Figure 14A**). The network revealed that TERT exhibits strong connections with other model genes, underscoring its role as a central hub within the network. Furthermore, we analyzed the expression of the 18 prognostic genes in tumor versus normal tissues from the TCGA database (**Supplementary Figure 14B**). The results demonstrated that TERT expression was significantly upregulated in tumor tissues, and its overexpression was associated with poorer overall survival in COAD patients from TCGA. To precisely determine the cellular localization of TERT, we incorporated single-cell and spatial transcriptomic data. Cell clustering and annotation of the GSE166555 dataset clearly visualized 13 distinct cell clusters following UMAP dimensionality reduction (**Figure 11A**). The UMAP visualization indicated that TERT expression was predominantly localized to malignant epithelial cells (**Figure 11B**). Quantification of TERT expression levels confirmed that its expression was significantly higher in tumor cells compared to all other cell types (**Figure 11C**). Cell subpopulations were categorized into TERT-positive and TERT-negative groups based on TERT expression status. The analysis revealed that TERT-positive tumor cells constituted a substantial majority (75.2%), whereas only 18.3% of cells in the TERT-negative group were tumor cells. This suggests that tumor cells are the primary contributors to TERT expression (**Figure 11D**). A consistent pattern was observed in spatial transcriptomic sections, where TERT was primarily expressed in tumor cell-enriched regions across 7 out of 10 sections (**Figure 11E**; **Supplementary Figure 15**). To further elucidate the biological processes and regulatory mechanisms through which TERT promotes tumor progression, cells in the GSE166555 single-cell dataset were similarly categorized into TERT-positive and TERT-negative groups. The AUCell package was used to score biological pathways related to immunity, metabolism, signaling, proliferation, cell death, and mitochondria, in order to assess pathway differences across cell types between TERT-positive and negative groups (**Figure 11F**). For the TCGA-COAD dataset, differential expression analysis was performed by comparing the 30% of samples with the highest TERT expression against the 30% with the lowest expression (**Figure 11G**). All genes from this analysis were ranked according to their log2FC values. GSEA was then conducted based on the Hallmark gene sets and KEGG metabolic gene sets. Comparing with the GSE166555 dataset, we observed that the E2F targets, G2/M checkpoint, and MYC signaling pathways were also significantly enriched in the TCGA-COAD cohort (**Figure 11H**). Proteins reported to directly interact with TERT were retrieved from the BioGRID database (https://thebiogrid.org) to construct a protein-protein interaction network (**Figure 11I**). Among these TERT-interacting proteins, five (BRCA2, CDK4, CDKN2A, MYC, TP53) are involved in the E2F pathway, seven (BRCA2, CDK4, DKC1, MYC, NCL, PML, UPF1) in the G2/M checkpoint, and five (APEX1, CDK4, MYC, PTGES3, YWHAQ) in the MYC pathway. This further supports TERT’s important role in inhibiting tumor cell apoptosis, regulating the cell cycle, and promoting proliferation.

### 3.11 Experimental verification

**Figure 12:**
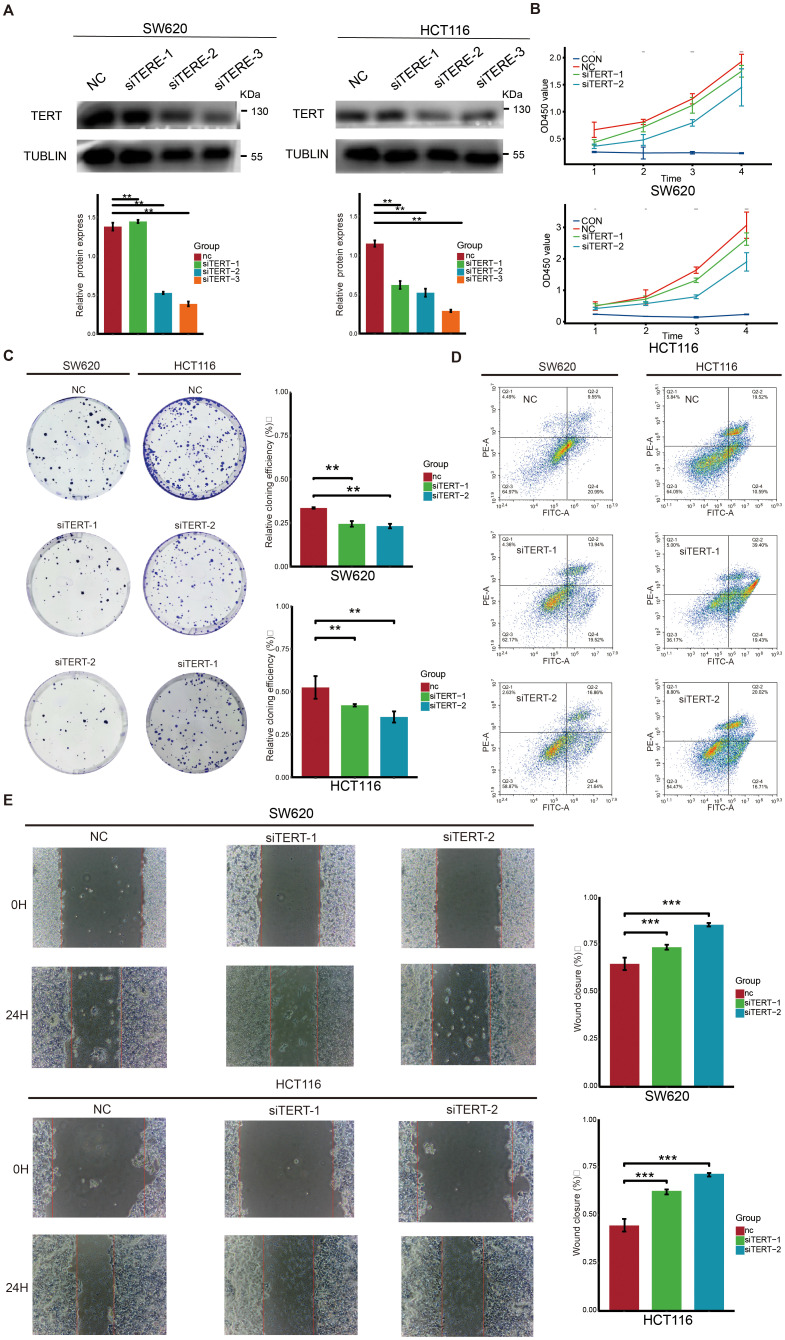
Cell experiment. **(A)** Western blot to evaluate the level of TERT expression 3 days after transfection and siRNA sequences could result in a significant decrease in TERT expression. **(B)** CCK8 assay showed that, after TERT knockdown, the cells showed a significant reduction in viability. **(C)** Colony formation assay displayed that cells with reduced TERT expression exhibited a significant reduction in the numbers of colonies. **(D)** Flow cytometry analysis showed that TERT knockdown significantly increased the percentage of apoptotic cells. **(E)** Scratch-wound healing assay depicted that a significantly slower wound healing rate was observed in cells with a decreased expression of TERT. All experiments were repeated in two COAD (SW620, HCT116) cell lines, and all data were presented as the means ± SD of three independent experiments. *P < 0.05, **P < 0.01, ***P < 0.001.

In order to further confirm these results, we performed functional studies *in vitro*. Initially, we evaluated the effectiveness of siRNA-mediated inhibition of TERT in HCT116 and SW620 cell lines by Western Blot analysis. Based on the Western Blot findings, it was determined that sequence 2 and sequence 3 exhibited a more effective knockdown effect. Therefore, these two sequences were selected for the further investigations (**Figure 12A**). The CCK-8 experiments demonstrated that suppressing TERT resulted in reduced proliferation of HCT116 and SW620 cells in comparison to the control group (**Figure 12B**). This suggests that TERT plays a role in facilitating the proliferation of COAD cell lines. The clonogenic tests provided evidence that the reduction of TERT expression impaired the capacity of COAD cells to form colonies (**Figure 12C**). Flow cytometry analysis further revealed that TERT knockdown significantly increased apoptosis in both cell lines (**Figures 12D**). Additionally, the wound healing assays revealed that the knockdown of TERT greatly hindered the migration and invasion of COAD cells (**Figure 12E**). To summarize, the tests above indicate that TERT acts as a pro-oncogenic regulator in the development and advancement of COAD tumors.

## 4 Discussion

It is evident that a multitude of genetic and epigenetic mechanisms, encompassing methylation, mutation, and histone modifications, are capable of regulating gene expression with a high degree of precision (44). Thus, a thorough examination of multi-omics data from patients allows us to get valuable knowledge on disease-specific regulatory processes (44, 45). Through the integration of 10 clustering algorithms, we have successfully identified five different prognostic subtypes in COAD patients. These subgroups possess unique characteristics that might potentially enhance the precision of stratified therapy for these patients. The stability of the new subtypes was established by many cohorts. It is also informative to consider how these five PCD-based subtypes relate to the well-established Consensus Molecular Subtypes (CMS) of colorectal cancer (46). The CS3 subtype, characterized by the best prognosis, the highest TMB, and robust immune infiltration, shares notable features with the MSI-immune CMS1 subtype, which is hypermutated, microsatellite unstable, and exhibits strong immune activation (47). The enrichment of cell cycle and senescence pathways in both CS2 and CS3 is reminiscent of the proliferative biology of CMS2 and CMS3, which are characterized by marked WNT and MYC signaling activation and metabolic dysregulation, respectively (46). Conversely, CS1, which exhibited relatively lower immune infiltration, may correspond in part to the mesenchymal and stroma-rich CMS4 subtype, which is characterized by prominent TGF-β activation, stromal invasion, and an immunosuppressive microenvironment (48). Although formal CMS classification was not performed in this study, these parallels suggest that PCD-based subtyping captures aspects of the biological diversity previously defined by transcriptome-wide consensus clustering, while potentially offering additional prognostic resolution through the integration of epigenetic and mutational dimensions, consistent with recent evidence that multi-omics-derived clusters can outperform CMS clusters in predicting patient survival (49).

Cell death is essential for preserving the overall balance of an organism, facilitating development, and avoiding the uncontrolled growth of cells (50, 51). Consequently, the full extent of the involvement of PCD patterns in COAD is still not well recognized. I n our study, we created a prognostic PCDS by combining the stepglm and Lasso methods in the TCGA dataset. Compared to existing PCD−based classifiers for colorectal cancer, our PCDS shows superior prognostic performance. The PCDS played a crucial role as an independent risk factor for the OS rate in COAD. The model demonstrated consistent and reliable performance in predicting patients’ OS rates. Furthermore, the ROC analysis demonstrated that the PCDS exhibited excellent accuracy and consistent performance across all five public GEO datasets. With the expertise of a pharmacologist, we integrated clinical information and developed a nomogram to improve the accuracy of our analysis. Our research revealed that the nomogram scores outperformed risk scores and other clinical characteristics in predicting survival.

The TME may have either beneficial or detrimental effects on tumor prognosis, depending on whether it promotes tumor elimination, increases tumor invasiveness, or enhances resistance to therapy (52). In order to comprehend how the TME affects tumor prognosis, immune cell infiltration was assessed in both high- and low-risk COAD patients in this research. Immune microenvironment analysis by ssGSEA, ESTIMATE, CIBERSORT, and MCP−counter consistently showed that the low−risk group has higher infiltration of CD8^+^ T cells, B cells, plasma cells, and myeloid dendritic cells, while the high−risk group is enriched for M0 macrophages and cancer−associated fibroblasts. Furthermore, the low-risk group exhibited elevated expression of most immune checkpoint genes, and correlation analysis revealed a significant inverse relationship between risk scores and the expression levels of these genes. Leveraging the resources of TCIA, we evaluated the response to anti-PD-1 and anti-CTLA-4 therapies to assess potential differences in treatment efficacy across distinct risk categories. The analysis revealed that the low-risk group exhibited significantly higher IPS values compared to the high-risk group, suggesting that COAD patients classified as low-risk are more likely to benefit from immunotherapy. Consistently, low−risk tumors also showed elevated TIGIT and TOX despite better prognosis; both molecules can be upregulated during early T cell activation and are not markers of terminal exhaustion, further supporting active antitumor immunity rather than dysfunction (53–56). Recent studies have applied subclass mapping algorithms to evaluate PD−1 and CTLA−4 treatment effects in colorectal cancer from the perspective of programmed cell death patterns, demonstrating that PCD−related signatures can effectively stratify patients likely to benefit from immunotherapy (57–59). Our CDS model thus offers a complementary tool to identify COAD patients who are most likely to respond to PD−1/CTLA−4 blockade, warranting prospective validation. This interpretation is further strengthened by the observation that the high−risk group harbors significantly higher aneuploidy scores, indicating greater genomic instability that may contribute to immune evasion and reduced immunotherapy response. Therefore, our PCDS risk score not only captures key prognostic information but also identifies COAD patients who are more likely to benefit from immune checkpoint blockade, warranting prospective clinical validation.

TMB’s significance in malignancies has been emphasized by recent research (60). High-risk patients showed increased mutation frequency in prognostically adverse genes, while low-risk patients had lower TMB. When stratified by median TMB and risk score, the H−TMB/low−risk subgroup had the most favorable prognosis, suggesting its potential utility for clinical risk stratification.

GSEA analysis showed that the high-risk group was mostly enriched in pathways related to autophagy, endocytosis, T cell receptor signaling pathway, and cell cycle. Tumor microenvironment and tumorigenesis are both significantly influenced by autophagy (61). Understanding the process of endocytosis is crucial in comprehending the various aspects of cancer, including drug stability, treatment response, and immune cell function (62).

Building upon these findings, our study integrates these layers of evidence to delineate a novel PCDS and underscore the pivotal role of TERT in COAD pathogenesis. Our identification of TERT requires careful interpretation. TERT is a known oncogene driving proliferation in colorectal cancer (16, 63); thus, its association with poor prognosis is not unexpected. The novelty lies in integrating TERT into a PCD−related multi−omics signature and, critically, demonstrating via single−cell and spatial transcriptomics that TERT is highly enriched within malignant tumor cells—a pattern conserved across dissociated cells and intact tissue sections. Pathway analyses link TERT to cell cycle pathways (E2F, G2/M, MYC) rather than a direct PCD mechanism. Therefore, TERT’s role in our model is likely indirect—promoting cell cycle dysregulation and apoptosis resistance—positioning it as a primary driver of the oncogenic phenotypes captured by our CDS. This contextualized interpretation within a broader cell death landscape underscores the value of integrating TERT into a PCD−related signature. Besides, we acknowledge that our study did not directly investigate cancer stem cell (CSC) markers. However, TERT, a core gene in our CDS, is highly expressed in CSCs and promotes self−renewal, DNA repair, and therapy resistance (64, 65). The enrichment of E2F, G2/M, and MYC pathways in the high−risk group is also characteristic of CSCs (66). These observations suggest a potential link between our PCD signature and CSC−driven aggressiveness, though direct validation is needed. Future studies should explore TERT−mediated stemness in COAD therapy resistance. Critically, our findings from single-cell and spatial transcriptomic analyses provide unprecedented resolution regarding the cellular basis of our observations. We unequivocally demonstrated that TERT expression is not ubiquitous but is highly enriched within malignant tumor cells, a pattern conserved across both dissociated single-cell suspensions and architecturally preserved tissue sections. This tumor cell-specific localization positions TERT as a primary driver of the oncogenic phenotypes we associated with the PCDS.

Several limitations of this study should be noted. First, the novel molecular subtypes and prognostic signature were constructed based on publicly available datasets and require further validation in larger, prospective clinical cohorts. Additionally, although our results suggest that the signature may serve as a prognostic biomarker and predictor of immunotherapy response, additional experimental and clinical studies are necessary to confirm these findings. Besides, the PCDS model showed modest and heterogeneous performance in external GEO cohorts, likely due to differences in patient composition, sample size, and platform technology; therefore, the current CDS should be considered a hypothesis−generating tool rather than a clinically ready classifier, and validation in uniformly collected prospective cohorts is warranted. Despite these limitations, our work identifies a new COAD subtype with distinct characteristics and elucidates the prognostic relevance and potential therapeutic implications of the PCDS through integrated machine learning approaches.

## 5 Conclusion

The work that we conducted used multi-omics consensus clustering to identify five molecular subtypes of COAD. These subtypes showed significant differences in prognosis, which could potentially improve the molecular classification of COAD. Subsequently, we constructed a prognostic model utilizing PCDS, which has the capability to forecast the efficacy of immunotherapy and offer innovative strategies for managing COAD. Additionally, our research indicates that TERT could serve as a promising target for therapeutic intervention in cases with COAD.

## Data Availability

Publicly available datasets were analyzed in this study. This data can be found here: Transcriptomic data, and clinical information of COAD in this study are available from the TCGA and GEO datasets.

## Supplementary material

Supplementary Figure 1 The distribution of each data set before and after batch correction through the PCA algorithm. **(A)** Before batch correction, **(B)** After batch correction.

Supplementary Figure 2 **(A)** The Cluster Prediction Index and Gap Statistical Analysis of the multiomics clusters. **(B)** The sample similarity of each subgroup was assessed by calculating the Silhoutte score.

Supplementary Figure 3 Differences between five chemotherapeutic agents Cisplatin **(A)**, Paclitaxel **(B)**, 5-Fluorouracl **(C)**, Gemcitabine **(D)**, Doxorubicin **(E)** in different subtypes of COAD.

Supplementary Figure 4 Survival analysis of hub genes in CDS (OS) in TCGA-COAD cohort.

Supplementary Figure 5 Comparison of C−index between our CDS and three published PCD−based signatures (PCDscore, MPCDI, PCDI) in TCGA−COAD.

Supplementary Figure 6 Repeated 10×5−fold cross−validation of the CDS model within TCGA−COAD.

Supplementary Figure 7 LASSO coefficient curves were selected with simulation parameters set to 1,000, and tenfold cross-validation of selecting tuning parameter in the LASSO model.

Supplementary Figure 8 **(A)** Contribution of PCD subtypes to the CDS model. **(B)** Risk coefficients of the 18CDS genes by PCD subtype.

Supplementary Figure 9 Multicox analysis for risk score and clinicopathological features of COAD patients in TCGA, GSE17536, GSE14333, GSE33133, GSE39582, GSE29621.

Supplementary Figure 10 **(A)** CIBERSORT immune cell fractions by risk group. **(B)** MCP−counter scores by risk group.

Supplementary Figure 11 Aneuploidy score distribution in high- vs. low-risk groups.

Supplementary Figure 12 Differential mutation frequencies of selected genes between high- and low-risk groups.

Supplementary Figure 13 Volcano map on differential genes between high- and low-risk groups.

Supplementary Figure 14 **(A)** A protein interaction network was explored by a string database. **(B)** Comparison of differential gene expression between tumor tissue and normal tissue of TCGA-COAD patients.

Supplementary Figure 15 **(A, B)** The sections were partitioned into distinct cellular microregions based on the most abundant cell type in the spatial transcriptomics data. **(C)** In the majority of sections, the spatial distribution of TERT expression was closely associated with tumor cells. **(D)** Spearman correlation analysis further revealed a significant positive correlation between TERT expression and tumor cells in most sections.

Supplementary Table 1 Oligonucleotides used in research.

Supplementary Table 2 Description of the 18 risk genes.

Supplementary Table 3 Comparison of effector/checkpoint ratios and exhaustion marker expression between low- and high-risk groups.

## References

[1] Bray F Laversanne M Sung H Ferlay J Siegel RL Soerjomataram I . Global cancer statistics 2022: GLOBOCAN estimates of incidence and mortality worldwide for 36 cancers in 185 countries. CA Cancer J Clin. (2024) 74:229–63. doi: 10.3322/caac.21834 38572751

[2] Kay Washington M . Colorectal carcinoma: Selected issues in pathologic examination and staging and determination of prognostic factors. Arch Pathol Lab Med. (2008) 132:1600–7. doi: 10.5858/2008-132-1600-CCSIIP 18834218

[3] Histological grading in colorectal cancer: New insights and perspectives. Histol Histopathol. (2015) 30:1059–67. doi: 10.14670/HH-11-633 26004398

[4] Biller LH Schrag D . Diagnosis and treatment of metastatic colorectal cancer: A review. JAMA. (2021) 325:669–85. doi: 10.1001/jama.2021.0106 33591350

[5] Miller KD Nogueira L Devasia T Mariotto AB Yabroff KR Jemal A . Cancer treatment and survivorship statistics, 2022. CA Cancer J Clin. (2022) 72:409–36. doi: 10.3322/caac.21731 35736631

[6] Qiao S Yang S Hua H Mao C Li X Cheng C . Identification of prognostic biomarkers in colorectal cancer through multi-omics profiling of programmed cell death pathways. J Gastrointest Oncol. (2025) 16:1503–20. doi: 10.21037/jgo-2024-861 40950352 PMC12432933

[7] Zhou Z Lin T Chen S Zhang G Xu Y Zou H . Omics-based molecular classifications empowering in precision oncology. Cell Oncol. (2024) 47:759–77. doi: 10.1007/s13402-023-00912-8 38294647 PMC12974067

[8] Wendt M Johanesen P Kang-Decker N Binion D Shah V Dwinell M . Silencing of epithelial CXCL12 expression by DNA hypermethylation promotes colonic carcinoma metastasis. Oncogene. (2006) 25:4986–97. doi: 10.1038/sj.onc.1209505 16568088 PMC4610155

[9] Taha SR Karimi M Mahdavi B Yousefi Tehrani M Bemani A Kabirian S . Crosstalk between non-coding RNAs and programmed cell death in colorectal cancer: Implications for targeted therapy. Epigenet Chromatin. (2025) 18:3. doi: 10.1186/s13072-024-00560-8 39810224 PMC11734566

[10] Waghela BN Vaidya FU Ranjan K Chhipa AS Tiwari BS Pathak C . AGE-RAGE synergy influences programmed cell death signaling to promote cancer. Mol Cell Biochem. (2021) 476:585–98. doi: 10.1007/s11010-020-03928-y 33025314

[11] Tower J . Programmed cell death in aging. Ageing Res Rev. (2015) 23:90–100. doi: 10.1016/j.arr.2015.04.002 25862945 PMC4480161

[12] Hanahan D . Hallmarks of cancer: New dimensions. Cancer Discov. (2022) 12:31–46. doi: 10.1158/2159-8290.CD-21-1059 35022204

[13] Galluzzi L Vitale I Aaronson SA Abrams JM Adam D Agostinis P . Molecular mechanisms of cell death: Recommendations of the nomenclature committee on cell death 2018. Cell Death Differ. (2018) 25:486–541. doi: 10.1038/s41418-017-0012-4 29362479 PMC5864239

[14] Zou Y Xie J Zheng S Liu W Tang Y Tian W . Leveraging diverse cell-death patterns to predict the prognosis and drug sensitivity of triple-negative breast cancer patients after surgery. Int J Surg. (2022) 107:106936. doi: 10.1016/j.ijsu.2022.106936 36341760

[15] Donega V Nijboer CH van Tilborg G Dijkhuizen RM Kavelaars A Heijnen CJ . Intranasally administered mesenchymal stem cells promote a regenerative niche for repair of neonatal ischemic brain injury. Exp Neurol. (2014) 261:53–64. doi: 10.1016/j.expneurol.2014.06.009 24945601

[16] Hanahan D Weinberg RA . Hallmarks of cancer: The next generation. Cell. (2011) 144:646–74. doi: 10.1016/j.cell.2011.02.013 21376230

[17] Patankar JV Becker C . Cell death in the gut epithelium and implications for chronic inflammation. Nat Rev Gastroenterol Hepatol. (2020) 17:543–56. doi: 10.1038/s41575-020-0326-4 32651553

[18] Dixon SJ Lemberg KM Lamprecht MR Skouta R Zaitsev EM Gleason CE . Ferroptosis: An iron-dependent form of nonapoptotic cell death. Cell. (2012) 149:1060–72. doi: 10.1016/j.cell.2012.03.042 22632970 PMC3367386

[19] Koren E Fuchs Y . Modes of regulated cell death in cancer. Cancer Discov. (2021) 11:245–65. doi: 10.1158/2159-8290.CD-20-0789 33462123

[20] Liao M Qin R Huang W Zhu HP Peng F Han B . Targeting regulated cell death (RCD) with small-molecule compounds in triple-negative breast cancer: A revisited perspective from molecular mechanisms to targeted therapies. J Hematol OncolJ Hematol Oncol. (2022) 15:1–44. doi: 10.1186/s13045-022-01260-0 35414025 PMC9006445

[21] Colaprico A Silva TC Olsen C Garofano L Cava C Garolini D . TCGAbiolinks: An R/Bioconductor package for integrative analysis of TCGA data. Nucleic Acids Res. (2016) 44:e71. doi: 10.1093/nar/gkv1507 26704973 PMC4856967

[22] Balar AV Galsky MD Rosenberg JE Powles T Petrylak DP Bellmunt J . Atezolizumab as first-line treatment in cisplatin-ineligible patients with locally advanced and metastatic urothelial carcinoma: A single-arm, multicentre, phase 2 trial. Lancet. (2017) 389:67–76. doi: 10.1016/S0140-6736(16)32455-2 27939400 PMC5568632

[23] Ritchie ME Phipson B Wu D Hu Y Law CW Shi W . limma powers differential expression analyses for RNA-sequencing and microarray studies. Nucleic Acids Res. (2015) 43:e47. doi: 10.1093/nar/gkv007 25605792 PMC4402510

[24] Cong P Wu T Huang X Liang H Gao X Tian L . Identification of the role and clinical prognostic value of target genes of m6A RNA methylation regulators in glioma. Front Cell Dev Biol. (2021) 9:709022. doi: 10.3389/fcell.2021.709022 34589481 PMC8473691

[25] Chen H Shi X Ren L . Identification of the miRNA-mRNA regulatory network associated with radiosensitivity in esophageal cancer based on integrative analysis of the TCGA and GEO data. BMC Med Genomics. (2022) 15:249. doi: 10.1186/s12920-022-01392-9 36456979 PMC9714096

[26] Wang Z Pan L Guo D . A novel five‐gene signature predicts overall survival of patients with hepatocellular carcinoma. Cancer Med. (2021) 10:3808–21. doi: 10.1002/cam4.3900 33934539 PMC8178492

[27] Wang X Peng W Li C Qin R Zhong Z Sun C . Identification of an immune-related signature indicating the dedifferentiation of thyroid cells. Cancer Cell Int. (2021) 21:231. doi: 10.1186/s12935-021-01939-3 33892730 PMC8067302

[28] Liu C Li X Shao H Li D . Identification and validation of two lung adenocarcinoma-development characteristic gene sets for diagnosing lung adenocarcinoma and predicting prognosis. Front Genet. (2020) 11:565206. doi: 10.3389/fgene.2020.565206 33408736 PMC7779611

[29] Wagner GP Kin K Lynch VJ . Measurement of mRNA abundance using RNA-seq data: RPKM measure is inconsistent among samples. Theory Biosci. (2012) 131:281–5. doi: 10.1007/s12064-012-0162-3 22872506

[30] Leek JT Johnson WE Parker HS Jaffe AE Storey JD . The sva package for removing batch effects and other unwanted variation in high-throughput experiments. Bioinforma Oxf Engl. (2012) 28:882–3. doi: 10.1093/bioinformatics/bts034 22257669 PMC3307112

[31] Johnson WE Li C Rabinovic A . Adjusting batch effects in microarray expression data using empirical Bayes methods. Biostatistics. (2007) 8:118–27. doi: 10.1093/biostatistics/kxj037 16632515

[32] Zhang X Shi M Chen T Zhang B . Characterization of the immune cell infiltration landscape in head and neck squamous cell carcinoma to aid immunotherapy. Mol Ther Nucleic Acids. (2020) 22:298–309. doi: 10.1016/j.omtn.2020.08.030 33230435 PMC7522342

[33] Huang M Liu L Zhu J . Identification of immune-related subtypes and characterization of tumor microenvironment infiltration in bladder cancer. Front Cell Dev Biol. (2021) 9:723817. doi: 10.3389/fcell.2021.723817 34532318 PMC8438153

[34] Lu X Meng J Su L . Multi‐omics consensus ensemble refines the classification of muscle‐invasive bladder cancer with stratified prognosis, tumour microenvironment and distinct sensitivity to frontline therapies. Clin Transl Med. (2021) 11:e601. doi: 10.1002/ctm2.601 34936229 PMC8693439

[35] Uhlitz F Bischoff P Peidli S . Mitogen‐activated protein kinase activity drives cell trajectories in colorectal cancer. EMBO Mol Med. (2021) 13:e14123. doi: 10.15252/emmm.202114123 34409732 PMC8495451

[36] Chalise P Fridley BL . Integrative clustering of multi-level ‘omic data based on non-negative matrix factorization algorithm. PloS One. (2017) 12:e0176278. doi: 10.1371/journal.pone.0176278 28459819 PMC5411077

[37] Tibshirani R Walther G Hastie T . Estimating the number of clusters in a data set via the gap statistic. J R Stat Soc Ser B Stat Methodol. (2001) 63:411–23. doi: 10.1111/1467-9868.00293 40046247

[38] Lu X Meng J Zhou Y Jiang L Yan F . MOVICS: An R package for multi-omics integration and visualization in cancer subtyping. Bioinformatics. (2021) 36:5539–41. doi: 10.1093/bioinformatics/btaa1018 33315104

[39] Peng Y Ouyang C Wu Y . A novel PCDscore based on programmed cell death-related genes can effectively predict prognosis and therapy responses of colon adenocarcinoma. Comput Biol Med. (2024) 170:107933. doi: 10.1016/j.compbiomed.2024.107933 38217978

[40] Li C Mao Y Liu Y . Machine learning-based integration develops a multiple programmed cell death signature for predicting the clinical outcome and drug sensitivity in colorectal cancer. Anticancer Drugs. (2025) 36:1–18. doi: 10.1097/CAD.0000000000001654 39132895

[41] Li J Jiang Y Nong S Liang L Chen L Gong Q . Development of a machine learning-derived programmed cell death index for prognostic prediction and immune insights in colorectal cancer. Discov Oncol. (2025) 16:608. doi: 10.1007/s12672-025-02323-7 40274671 PMC12021754

[42] Lai C Wu Z Li Z . A robust signature of immune‐related long non‐coding RNA to predict the prognosis of bladder cancer. Cancer Med. (2021) 10:6534–45. doi: 10.1002/cam4.4167 34374227 PMC8446409

[43] Chang TG Cao Y Shulman ED Ben-David U Schäffer AA Ruppin E . Optimizing cancer immunotherapy response prediction by tumor aneuploidy score and fraction of copy number alterations. NPJ Precis Oncol. (2023) 7:54. doi: 10.1038/s41698-023-00408-6 37270587 PMC10239491

[44] Oh M Park S Kim S Chae H . Machine learning-based analysis of multi-omics data on the cloud for investigating gene regulations. Brief Bioinform. (2021) 22:66–76. doi: 10.1093/bib/bbaa032 32227074

[45] Reel PS Reel S Pearson E Trucco E Jefferson E . Using machine learning approaches for multi-omics data analysis: A review. Biotechnol Adv. (2021) 49:107739. doi: 10.1016/j.bioteChadv.2021.107739 33794304

[46] Guinney J Dienstmann R Wang X . The consensus molecular subtypes of colorectal cancer. Nat Med. (2015) 21:1350–6. doi: 10.1038/nm.3967 26457759 PMC4636487

[47] Chowdhury S Xiu J Ribeiro JR . Consensus molecular subtyping of metastatic colorectal cancer expands biomarker-directed therapeutic benefit for patients with CMS1 and CMS2 tumors. Br J Cancer. (2024) 131:1328–39. doi: 10.1038/s41416-024-02826-0 39227409 PMC11473766

[48] Leonard NA Corry SM Reidy E . Tumor-associated mesenchymal stromal cells modulate macrophage phagocytosis in stromal-rich colorectal cancer via PD-1 signaling. iScience. (2024) 27:110701. doi: 10.1016/j.isci.2024.110701 39310770 PMC11416555

[49] Ma Y Li J Zhao X . Multi-omics cluster defines the subtypes of CRC with distinct prognosis and tumor microenvironment. Eur J Med Res. (2024) 29:207. doi: 10.1186/s40001-024-01805-8 38549156 PMC10976740

[50] Bertheloot D Latz E Franklin BS . Necroptosis, pyroptosis and apoptosis: An intricate game of cell death. Cell Mol Immunol. (2021) 18:1106–21. doi: 10.1038/s41423-020-00630-3 33785842 PMC8008022

[51] Strasser A Vaux DL . Cell death in the origin and treatment of cancer. Mol Cell. (2020) 78:1045–54. doi: 10.1016/j.molcel.2020.05.014 32516599

[52] Swartz MA Iida N Roberts EW . Tumor microenvironment complexity: Emerging roles in cancer therapy. Cancer Res. (2012) 72:2473–80. doi: 10.1158/0008-5472.CAN-12-0122 22414581 PMC3653596

[53] Niu H Wang H . TOX regulates T lymphocytes differentiation and its function in tumor. Front Immunol. (2023) 14:990419. doi: 10.3389/fimmu.2023.990419 36969216 PMC10035881

[54] Scott AC Dündar F Zumbo P . TOX is a critical regulator of tumour-specific T cell differentiation. Nature. (2019) 571:270–4. doi: 10.1038/s41586-019-1324-y 31207604 PMC7698992

[55] Hu F Wang W Fang C Bai C . TIGIT presents earlier expression dynamic than PD-1 in activated CD8+ T cells and is upregulated in non-small cell lung cancer patients. Exp Cell Res. (2020) 396:112260. doi: 10.1016/j.yexcr.2020.112260 32890458

[56] Banta KL Xu X Chitre AS . Mechanistic convergence of the TIGIT and PD-1 inhibitory pathways necessitates co-blockade to optimize anti-tumor CD8+ T cell responses. Immunity. (2022) 55:512–526.e9. doi: 10.1016/j.immuni.2022.02.005 35263569 PMC9287124

[57] Gu X Pan J Li Y Feng L . A programmed cell death-related gene signature to predict prognosis and therapeutic responses in liver hepatocellular carcinoma. Discov Oncol. (2024) 15:71. doi: 10.1007/s12672-024-00924-2 38466483 PMC10928056

[58] Charoentong P Finotello F Angelova M . Pan-cancer immunogenomic analyses reveal genotype-immunophenotype relationships and predictors of response to checkpoint blockade. Cell Rep. (2017) 18:248–62. doi: 10.1016/j.celrep.2016.12.019 28052254

[59] Jiang P Gu S Pan D . Signatures of T cell dysfunction and exclusion predict cancer immunotherapy response. Nat Med. (2018) 24:1550–8. doi: 10.1038/s41591-018-0136-1 30127393 PMC6487502

[60] Chan TA Yarchoan M Jaffee E . Development of tumor mutation burden as an immunotherapy biomarker: Utility for the oncology clinic. Ann Oncol. (2019) 30:44–56. doi: 10.1093/annonc/mdy495 30395155 PMC6336005

[61] Debnath J Gammoh N Ryan KM . Autophagy and autophagy-related pathways in cancer. Nat Rev Mol Cell Biol. (2023) 24(8):560–75. doi: 10.1038/s41580-023-00585-z 36864290 PMC9980873

[62] Banushi B Joseph SR Lum B Lee JJ Simpson F . Endocytosis in cancer and cancer therapy. Nat Rev Cancer. (2023) 23:450–73. doi: 10.1038/s41568-023-00574-6 37217781

[63] Shay JW Wright WE . Telomeres and telomerase in normal and cancer stem cells. FEBS Lett. (2010) 584:3819–25. doi: 10.1016/j.febslet.2010.05.026 20493857 PMC3370416

[64] Akıncılar SC Chua JYH Ng QF . Identification of mechanism of cancer-cell-specific reactivation of hTERT offers therapeutic opportunities for blocking telomerase specifically in human colorectal cancer. Nucleic Acids Res. (2023) 51:1–16. doi: 10.1093/nar/gkac479 35697349 PMC9841410

[65] Liu Y Betori RC Pagacz J . Targeting telomerase reverse transcriptase with the covalent inhibitor NU-1 confers immunogenic radiation sensitization. Cell Chem Biol. (2022) 29:1517–1531.e7. doi: 10.1016/j.chembiol.2022.09.002 36206753 PMC9588800

[66] Li Z Yang Z Liu W . Disheveled3 enhanced EMT and cancer stem-like cells properties via Wnt/β-catenin/c-Myc/SOX2 pathway in colorectal cancer. J Transl Med. (2023) 21:302. doi: 10.1186/s12967-023-04120-8 37147666 PMC10161491

