# Supplementary figures and images for: Molecular subtyping and prognostic modeling of colon adenocarcinoma based on programmed cell death features: a multi-omics and machine learning study
Source: Front Immunol. 2026 Jun 11;17:1736554. doi: 10.3389/fimmu.2026.1736554 (PMC13294056; doi:10.3389/fimmu.2026.1736554)

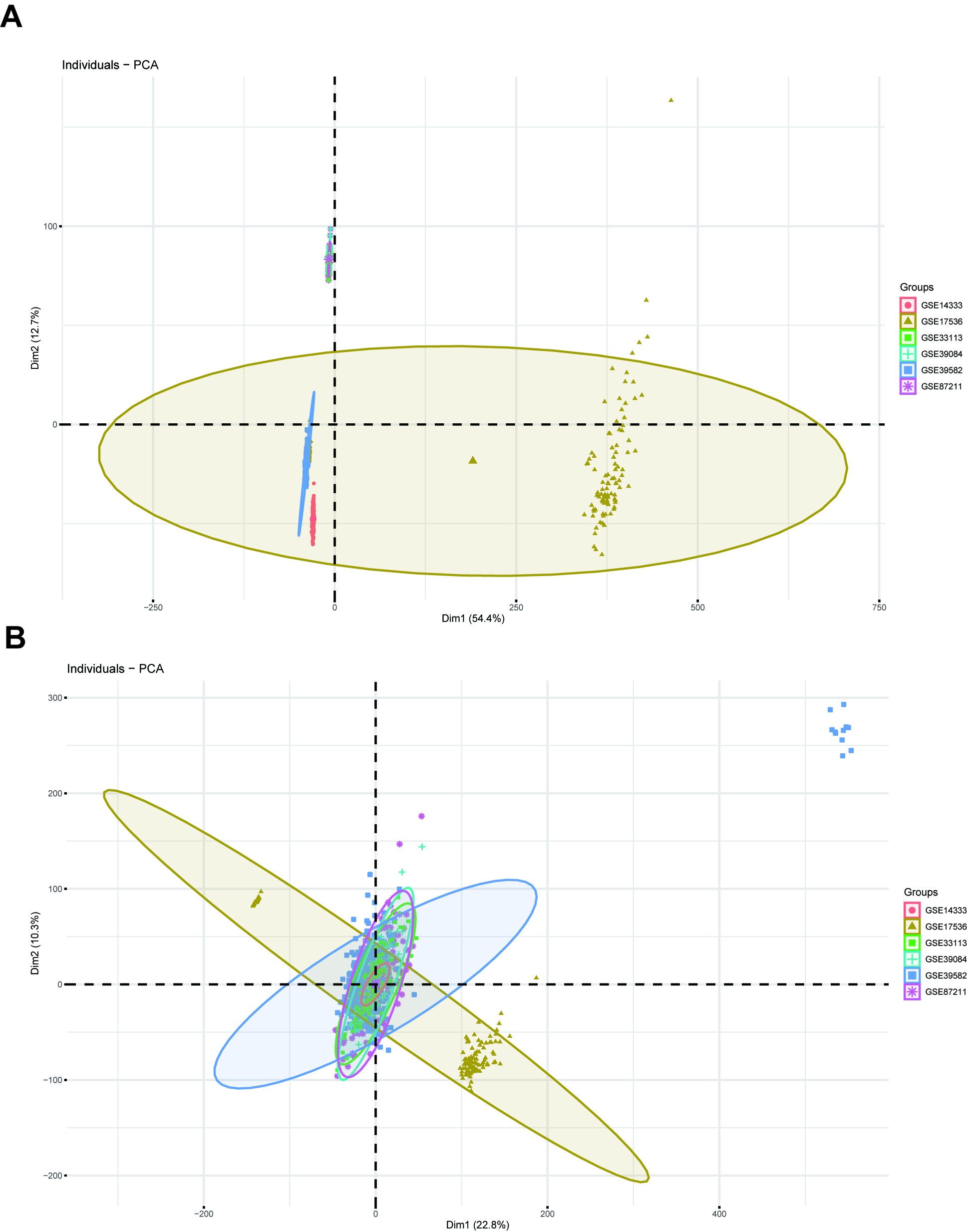

Supplement: Supplementary Figure 1 — The distribution of each data set before and after batch correction through the PCA algorithm. (A) Before batch correction, (B) After batch correction. [file Image1.tif]

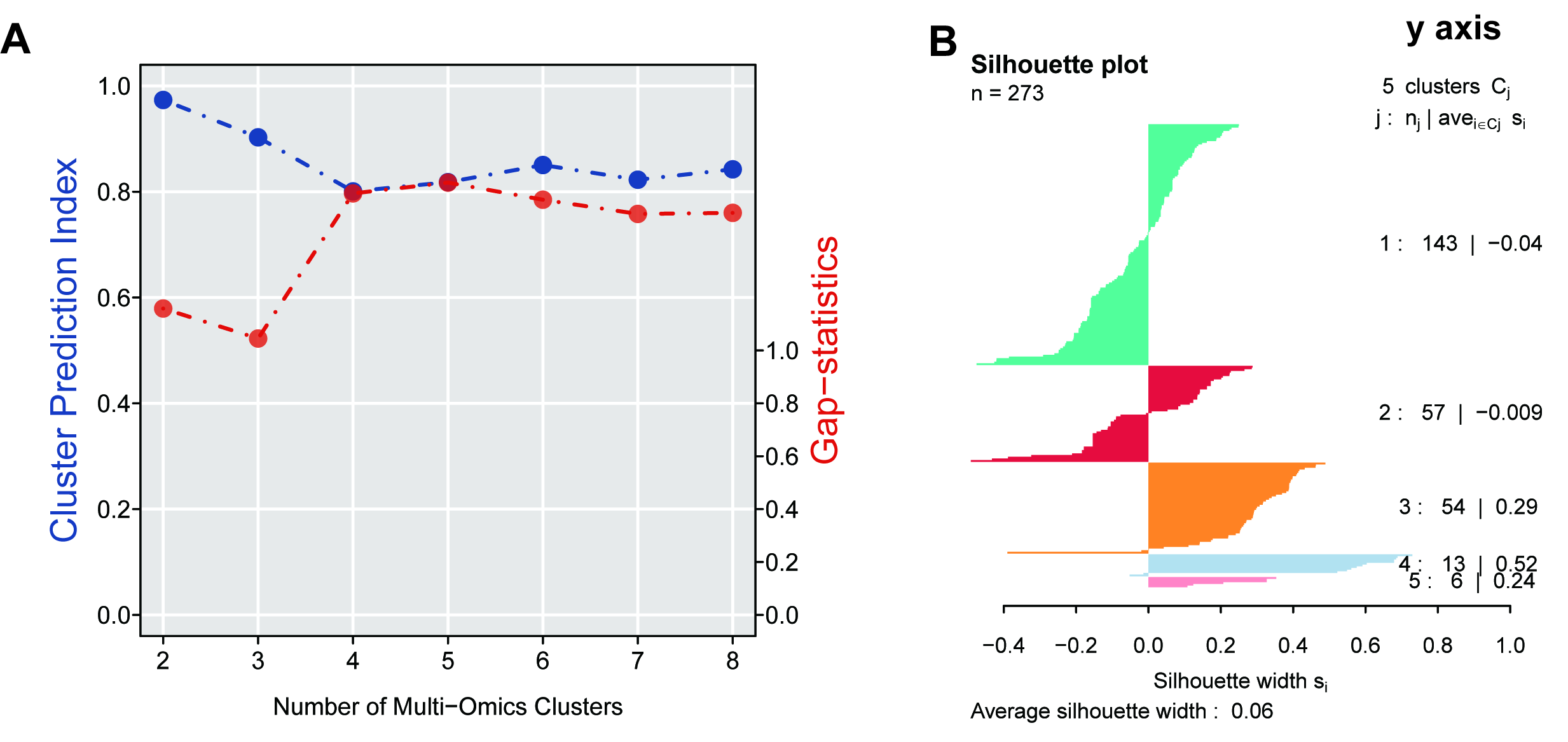

Supplement: Supplementary Figure 2 — (A) The Cluster Prediction Index and Gap Statistical Analysis of the multiomics clusters. (B) The sample similarity of each subgroup was assessed by calculating the Silhoutte score. [file Image2.tif]

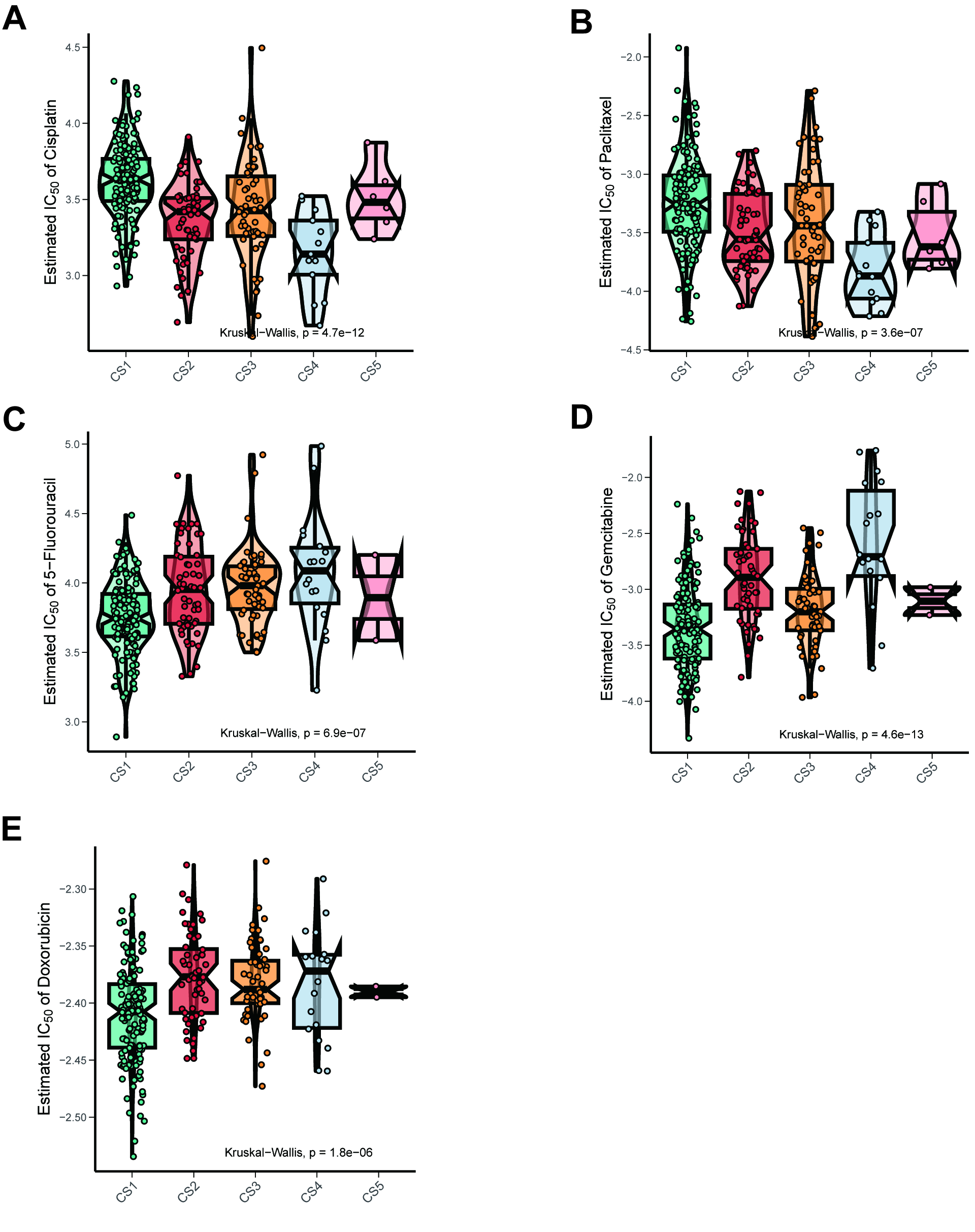

Supplement: Supplementary Figure 3 — Differences between five chemotherapeutic agents Cisplatin (A), Paclitaxel (B), 5-Fluorouracl (C), Gemcitabine (D), Doxorubicin (E) in different subtypes of COAD. [file Image3.tif]

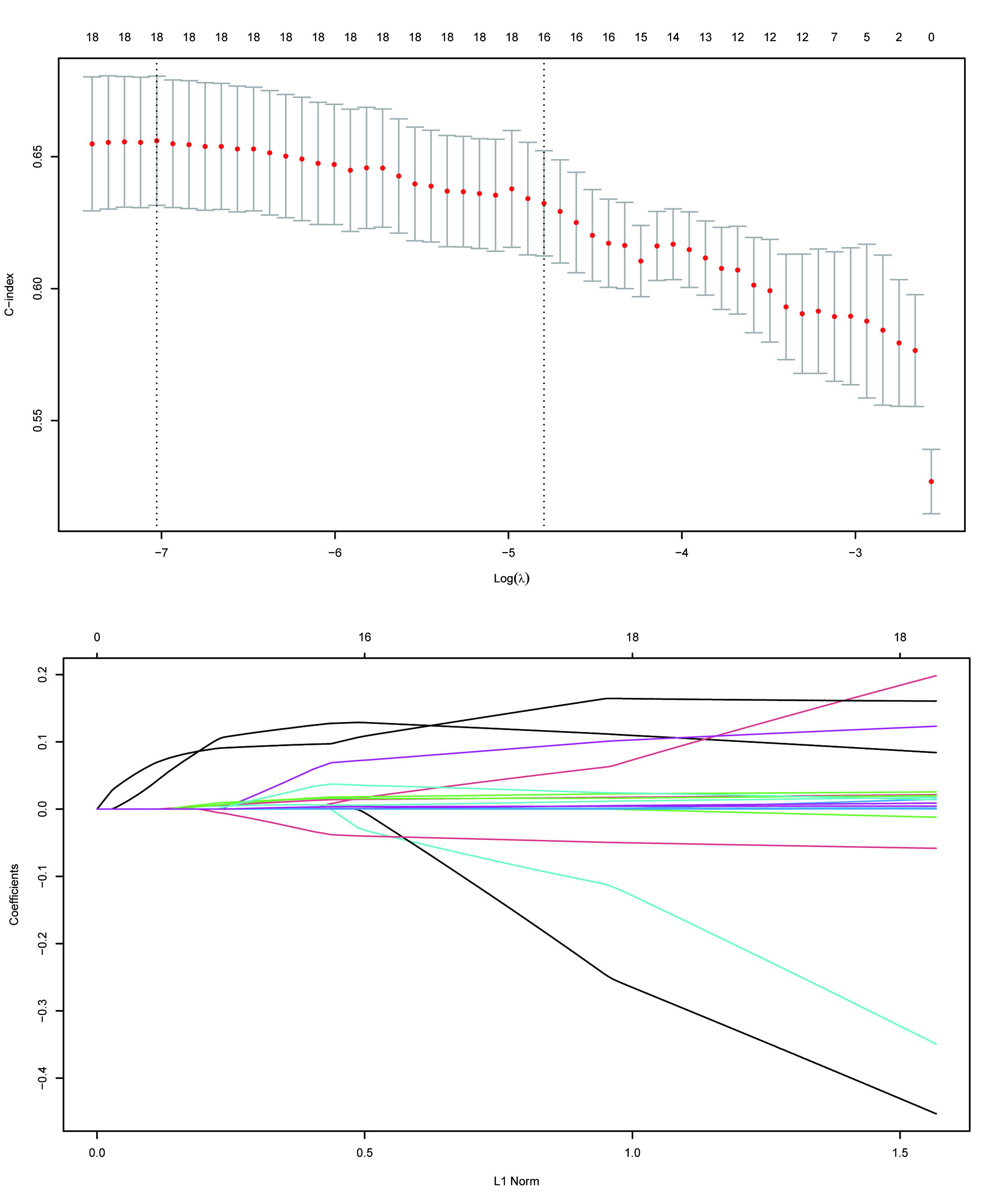

Supplement: Supplementary Figure 4 — Survival analysis of hub genes in CDS (OS) in TCGA-COAD cohort. [file Image4.tif]

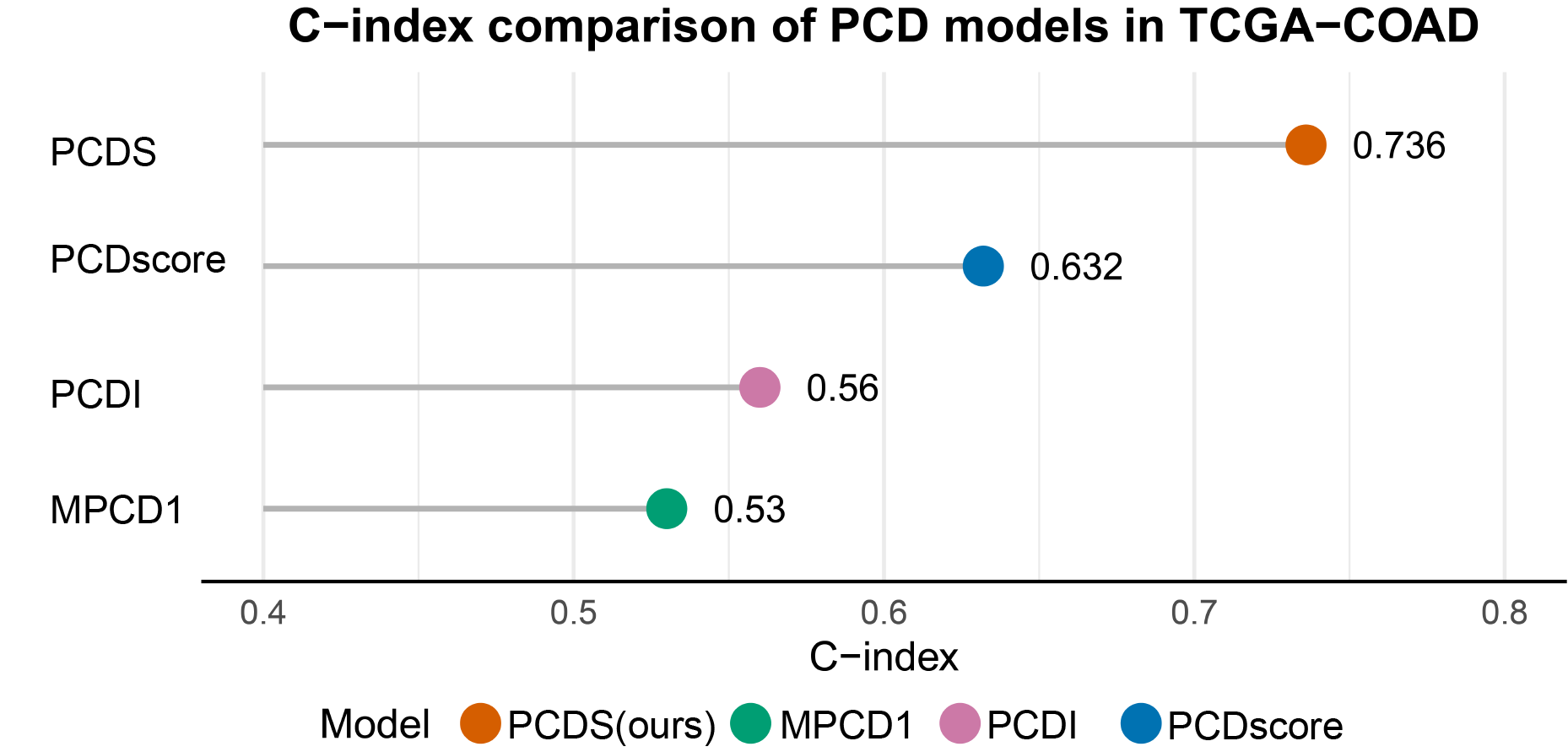

Supplement: Supplementary Figure 5 — Comparison of C−index between our CDS and three published PCD−based signatures (PCDscore, MPCDI, PCDI) in TCGA−COAD. [file Image5.tif]

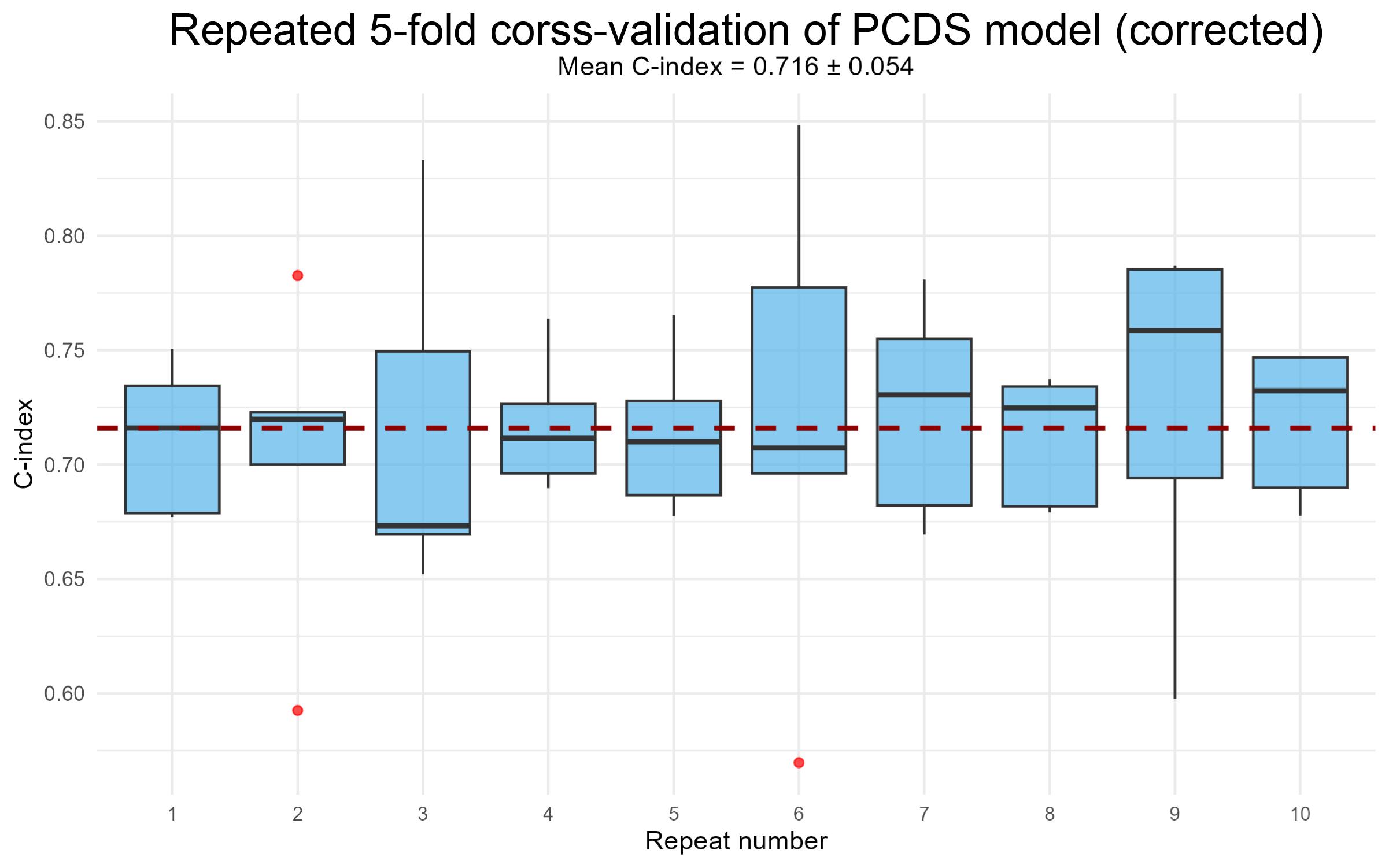

Supplement: Supplementary Figure 6 — Repeated 10×5−fold cross−validation of the CDS model within TCGA−COAD. [file Image6.tif]

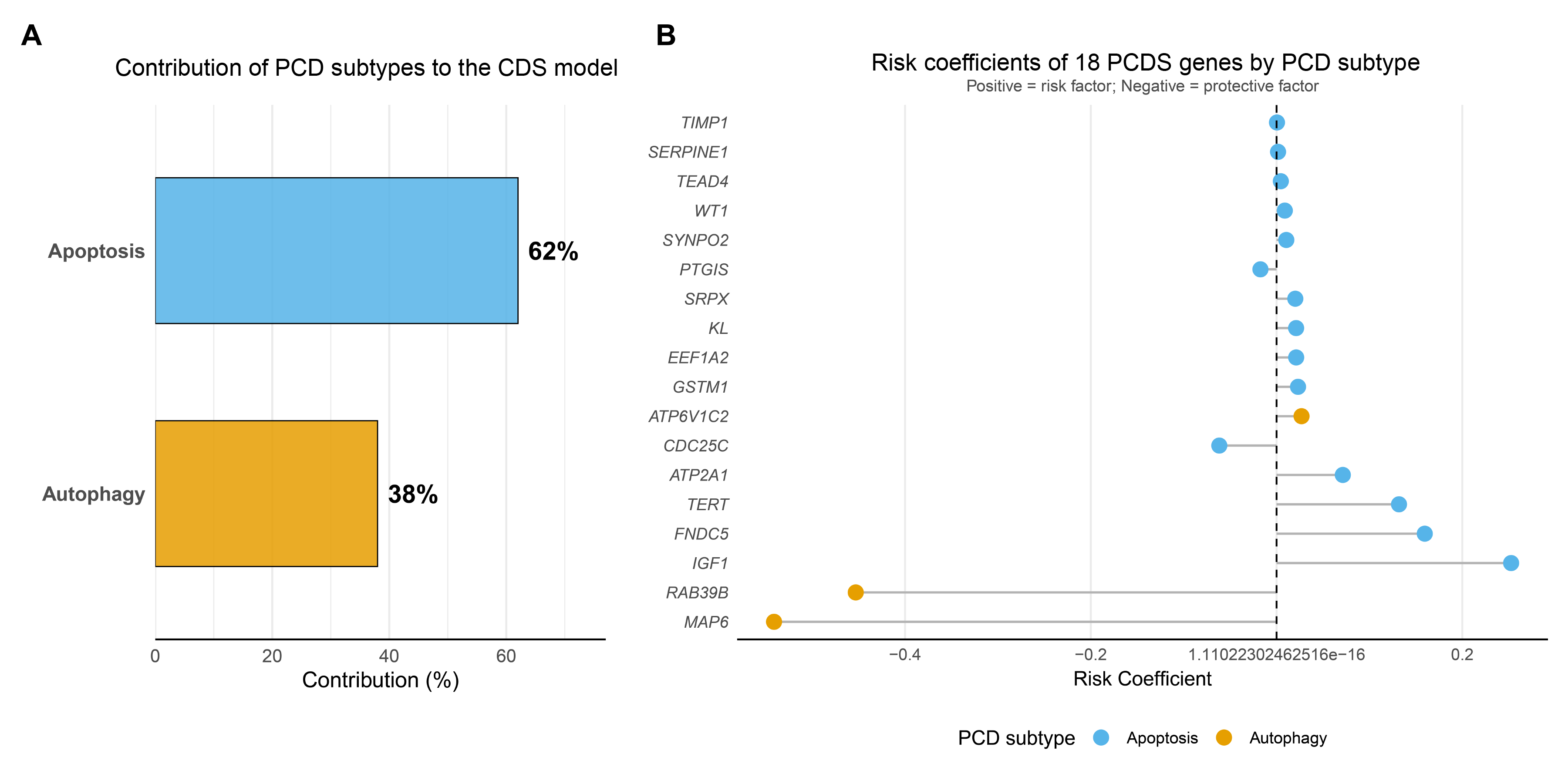

Supplement: Supplementary Figure 8 — (A) Contribution of PCD subtypes to the CDS model. (B) Risk coefficients of the 18CDS genes by PCD subtype. [file Image8.tif]

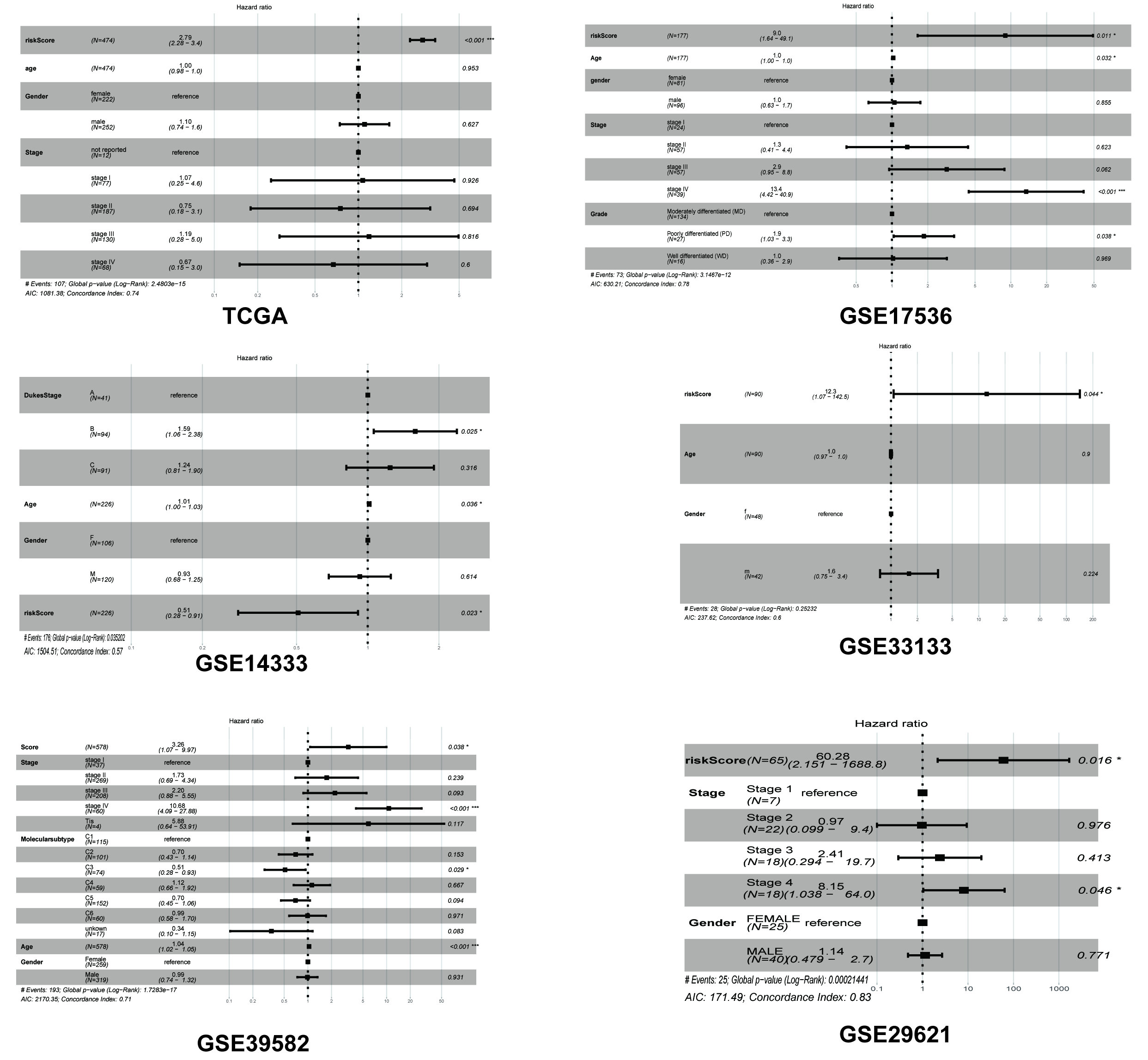

Supplement: Supplementary Figure 9 — Multicox analysis for risk score and clinicopathological features of COAD patients in TCGA, GSE17536, GSE14333, GSE33133, GSE39582, GSE29621. [file Image9.tif]

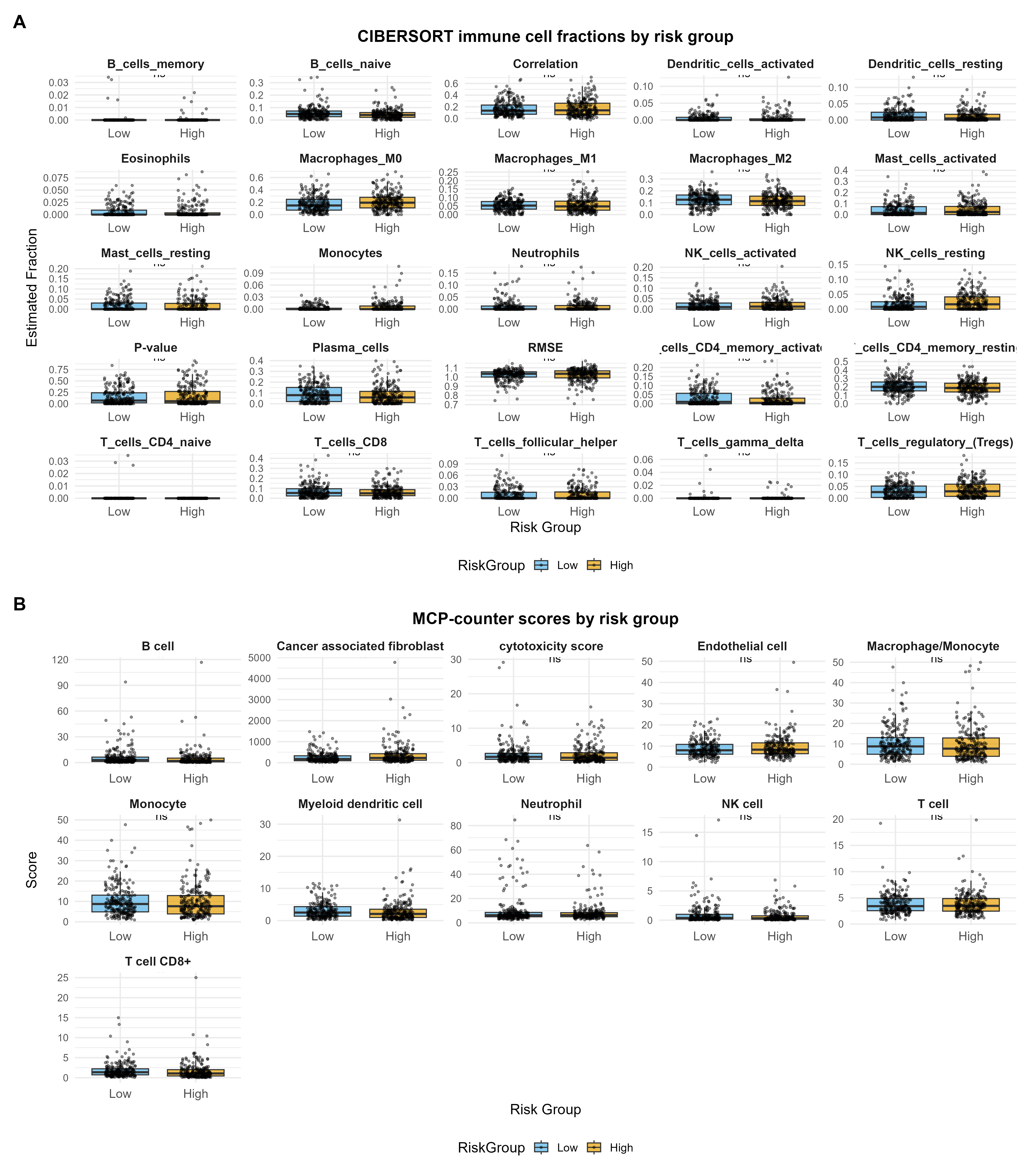

Supplement: Supplementary Figure 10 — (A) CIBERSORT immune cell fractions by risk group. (B) MCP−counter scores by risk group. [file Image10.tif]

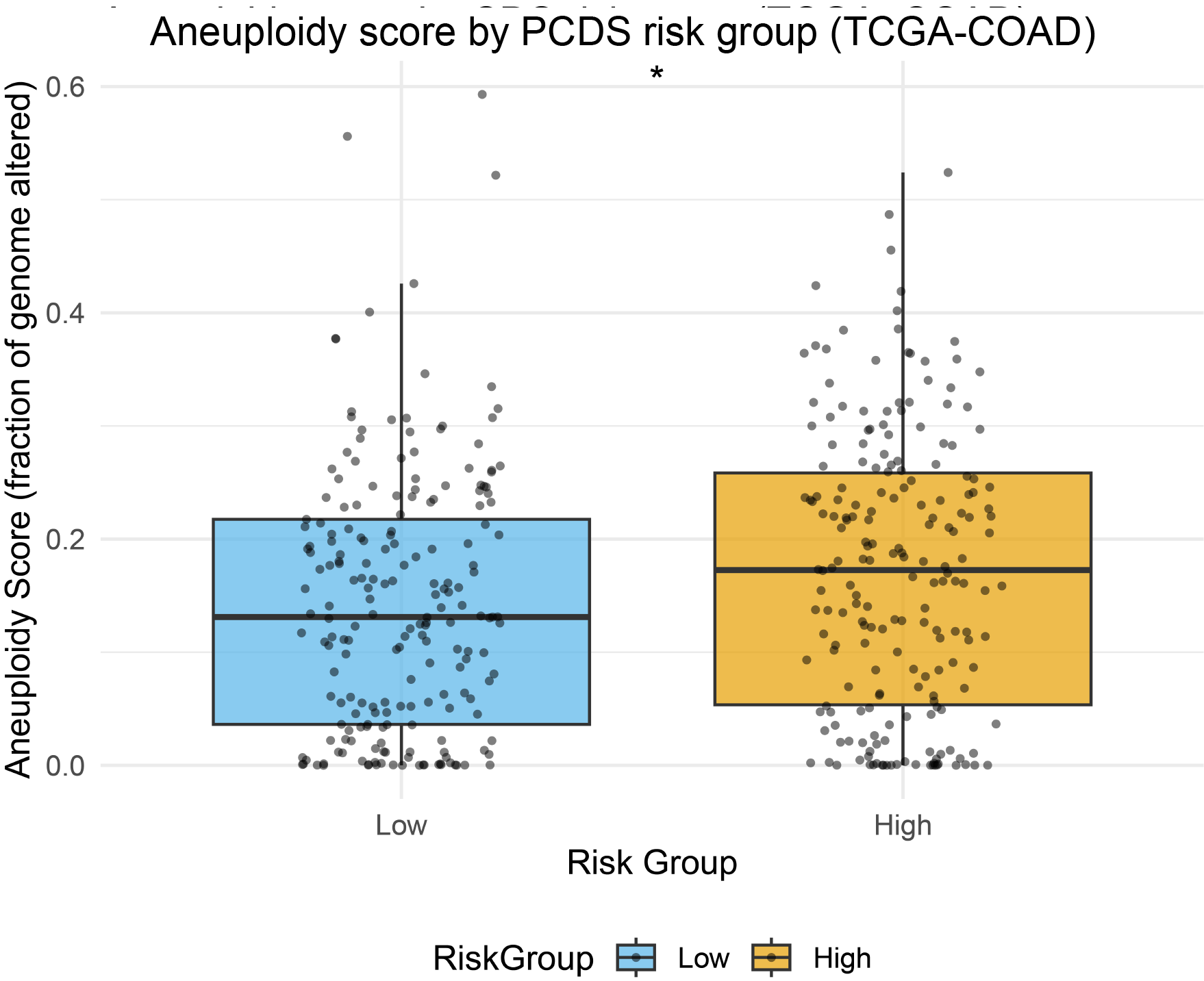

Supplement: Supplementary Figure 11 — Aneuploidy score distribution in high- vs. low-risk groups. [file Image11.tif]

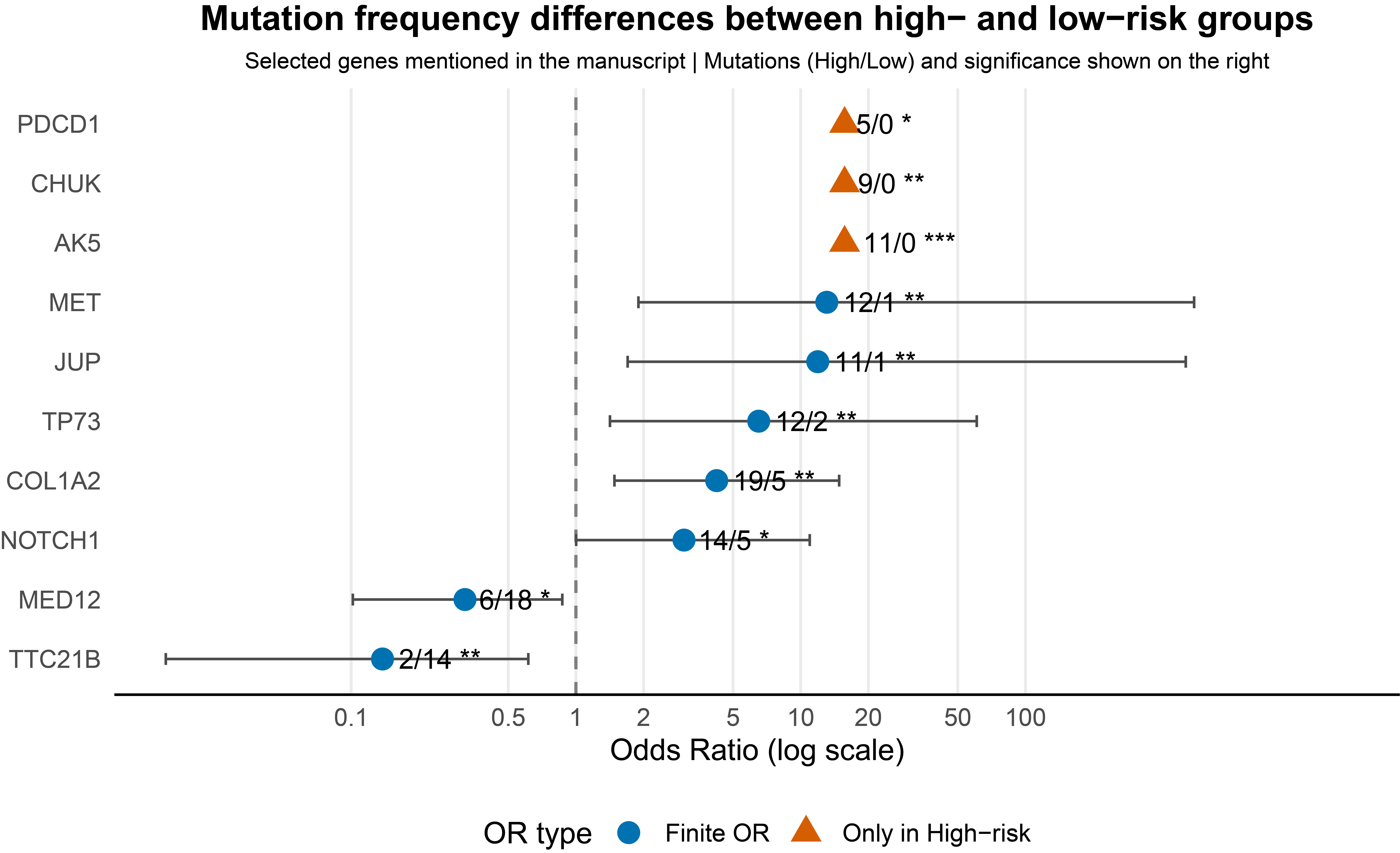

Supplement: Supplementary Figure 12 — Differential mutation frequencies of selected genes between high- and low-risk groups. [file Image12.tif]

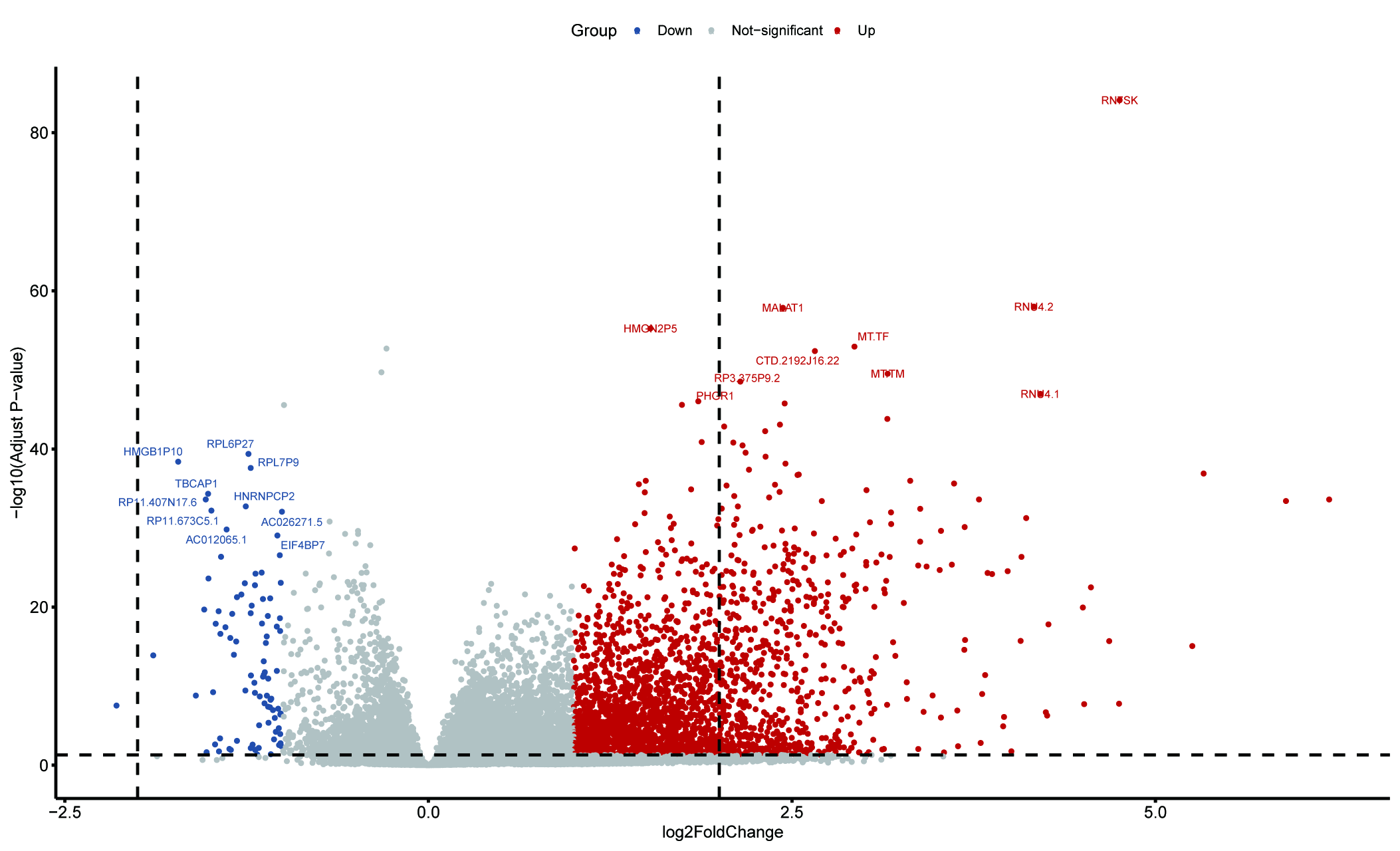

Supplement: Supplementary Figure 13 — Volcano map on differential genes between high- and low-risk groups. [file Image13.tif]

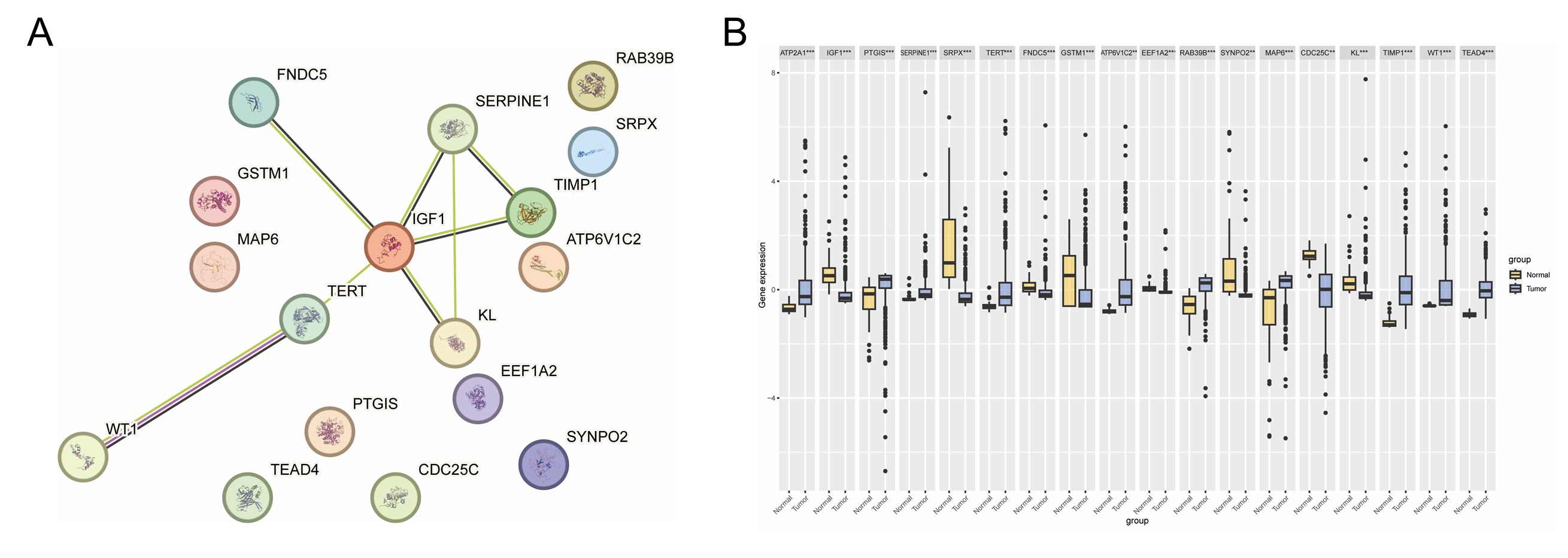

Supplement: Supplementary Figure 14 — (A) A protein interaction network was explored by a string database. (B) Comparison of differential gene expression between tumor tissue and normal tissue of TCGA-COAD patients. [file Image14.tif]

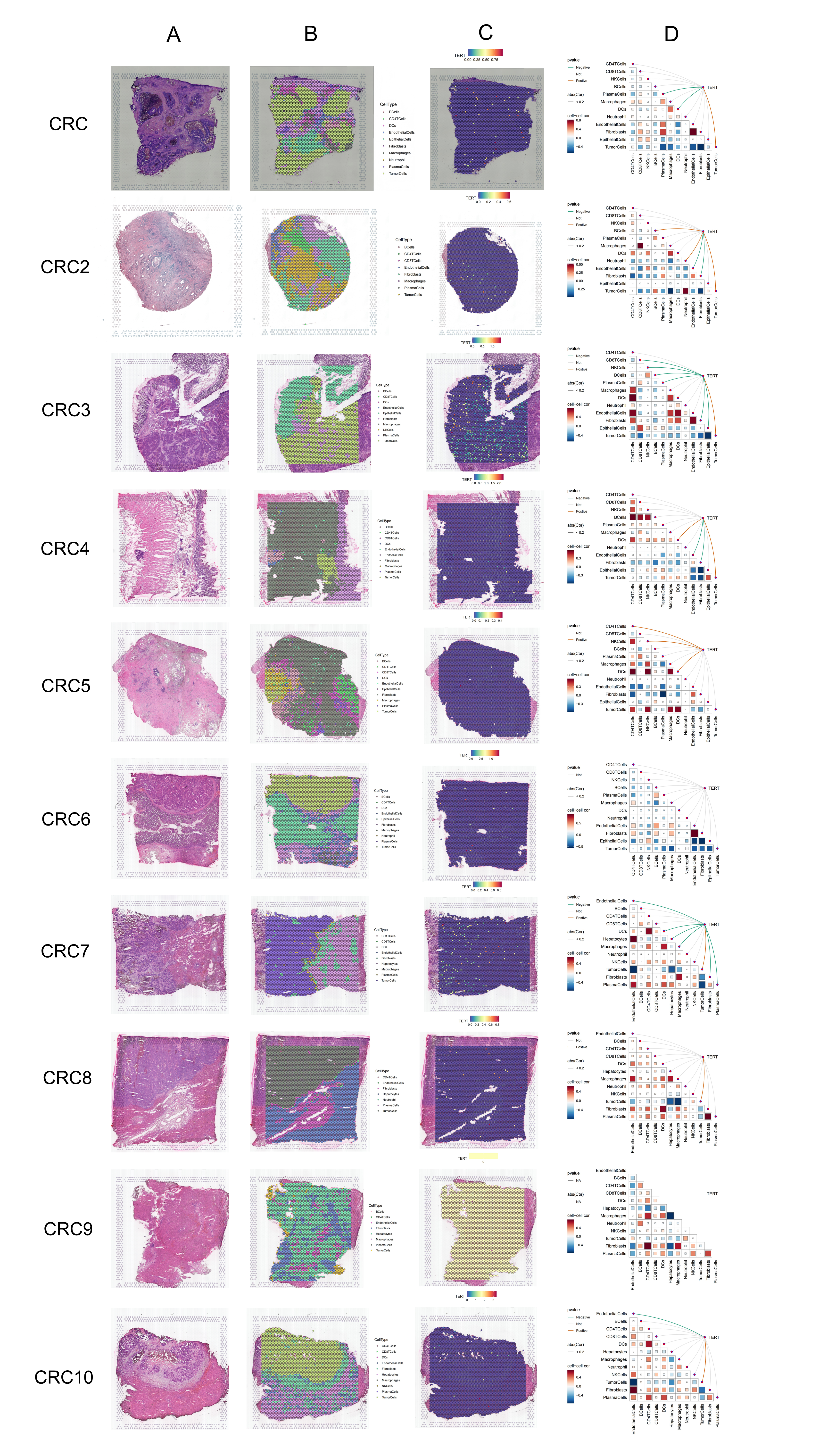

Supplement: Supplementary Figure 15 — (A, B) The sections were partitioned into distinct cellular microregions based on the most abundant cell type in the spatial transcriptomics data. (C) In the majority of sections, the spatial distribution of TERT expression was closely associated with tumor cells. (D) Spearman correlation analysis further revealed a significant positive correlation between TERT expression and tumor cells in most sections. [file Image15.tif]
