# Supplementary material for: Molecular subtyping and prognostic modeling of colon adenocarcinoma based on programmed cell death features: a multi-omics and machine learning study
Source: Front Immunol. 2026 Jun 11;17:1736554. doi: 10.3389/fimmu.2026.1736554 (PMC13294056; doi:10.3389/fimmu.2026.1736554)
Supplement: Supplementary Table 2 — Description of the 18 risk genes. [file Table2.docx]

| SYMBOL | ENSEMBL | Description |
| --- | --- | --- |
| ATPA2 | ENSG00000174437 | ATPase Sarcoplasmic/Endoplasmic Reticulum Ca2+ |
| IGF1 | ENSG00000017427 | Insulin Like Growth Factor 1 |
| PTGIS | ENSG00000124212 | Prostaglandin I2 Synthase |
| SERPINE1 | ENSG00000106366 | Serpin Family E Member 1 |
| SRPX | ENSG00000101955 | Sushi Repeat Containing Protein X-Linked |
| TERT | ENSG00000164362 | Telomerase Reverse Transcriptase |
| FNDC5 | ENSG00000160097 | Fibronectin Type III Domain Containing 5 |
| GSTM1 | ENSG00000134184 | Glutathione S-Transferase Mu 1 |
| ATP6V1C2 | ENSG00000143882 | ATPase H+ Transporting V1 Subunit C2 |
| EEF1A2 | ENSG00000101210 | Eukaryotic Translation Elongation Factor 1 Alpha 2 |
| RAB39B | ENSG00000155961 | RAB39B, Member RAS Oncogene Family |
| SYNPO2 | ENSG00000172403 | Synaptopodin 2 |
| MAP6 | ENSG00000171533 | Microtubule Associated Protein 6 |
| CDC25C | ENSG00000158402 | Cell Division Cycle 25C |
| KL | ENSG00000133116 | Klotho |
| TIMP1 | ENSG00000102265 | TIMP Metallopeptidase Inhibitor 1 |
| WT1 | ENSG00000184937 | WT1 Transcription Factor |
| TEAD4 | ENSG00000197905 | TEA Domain Transcription Factor 4 |

Table S2 Description of the 18 risk genes.
